# The burden of alcoholic cardiomyopathy in China and different regions around the world

**DOI:** 10.7189/jogh.12.04041

**Published:** 2022-07-30

**Authors:** Jing Zhang, Hailing Liu, Xiang Bu, Qun Lu, Lu Cheng, Aiqun Ma, Tingzhong Wang

**Affiliations:** 1 Department of Cardiovascular Medicine, The First Affiliated Hospital of Xi’an Jiaotong University, Xi’an, Shaanxi, China; 2 Key Laboratory of Molecular Cardiology, Xi’an, Shaanxi, China; 3 Key Laboratory of Environment and Genes Related to Diseases, Xi’an Jiaotong University, Ministry of Education, China; 4 Department of Pediatrics, The First Affiliated Hospital of Xi’an Jiaotong University, Xi’an, Shaanxi, China; 5 Department of Respiratory and Critical Care Medicine, The First Affiliated Hospital of Xi’an Jiaotong University, Xi’an, Shaanxi, China; 6 Department of Cardiovascular Medicine, The Affiliated Cardiovascular Hospital of Qingdao University, Qingdao, Shandong, China

## Abstract

**Background:**

Alcoholic cardiomyopathy (ACM) remains a significant public health issue with a growing global burden. The burden of ACM in China and different regions remains poorly understood.

**Methods:**

Data on ACM deaths, disability-adjusted life years (DALYs), the corresponding global age-standardized death rate (ASDR), age-standardized DALY rate and estimated annual percentage change (EAPC) were analysed based on age, sex, socio-demographic index (SDI) quintiles, different regions and in China from the Global Burden of Disease (GBD) study 2019.

**Results:**

Globally, the death rate and DALYs due to ACM were 71 723 and 2 441 108 in 2019, 33.06% and 38.79% increase from 1990, respectively. The corresponding ASDR and age-standardized DALY rate decreased with EAPC of -1.52 (95% uncertainty interval (UI) = -2.39, -0.65) and -1.12 (95% UI = -2.14, -0.10). The high-middle SDI regions, especially Eastern Europe, showed the highest number of ACM-related deaths and DALYs. The ACM-related deaths and DALYs were 2545 and 87823 in China in 2019, 171.03% and 147.17% increase from 1990, respectively. Unlike the world level, ASDR and age-standardized DALY rate also increased in China. The ACM burden is higher in men, and people with 50 to 69 years old accounted for the most.

**Conclusions:**

ACM burden in China and across the world increased substantially from 1990 to 2019. The greatest burden was borne by the high-middle SDI regions, especially by men aged 50-69 years old. Geographically and gender-age tailored strategies were needed to prevent ACM.

Alcoholic cardiomyopathy (ACM), first described in 1877 [1], is a myocardial disease caused by long-term heavy alcohol consumption. It is characterized by impaired ventricular dilation and contractility with normal or reduced ventricular wall thickness [2,3]. Alcohol abuse is an important cause of non-ischemic cardiomyopathy, accounting for 10% cases of dilated cardiomyopathies [4]. Extensive studies have proved the cardiotoxic effects of alcohol and its metabolite on cardiotoxicity [5].

Ethanol intoxication caused by excessive alcohol consumption is closely associated with myocardial injury. The mechanisms are as follows. First, ethanol can disrupt the composition and permeability of the plasma membrane, myogenic fibres and intermediate filament bridge grain contacts, leading to structural cell instability [6]. Second, ethanol may interfere with signal transduction mechanisms, interfere with the electrophysiological properties of ion channels such as Na^+^ and K^+^ channels [7,8], L-type calcium channels [9,10] and Na^+^/K^+^ ATPase [11], thereby disrupting excitation-contraction coupling and decreasing cardiac contractility. Third, ethanol can affect the synthesis of cardiac proteins [12]. Finally, chronic high-dose alcohol consumption induces apoptosis in cardiomyocytes through the BAX and BCL-2 pathways [13].

Globally, 32.5% of population are current drinkers, and alcohol consumption per capita in the world population increased from 5.5 L of alcohol in 2005 to 6.4 L in 2010 [14]. The occurrence of ACM is strongly related to the dose and frequency of alcohol consumption. However, there is no threshold for alcohol consumption that would lead to the development of ACM [15,16]. It has been suggested that the policies targeting the total consumption level of the population are needed to reduce the burden of ACM globally [17].

According to the 2019 Global Burden of Disease (GBD) study, we analysed the current condition and temporal trends of fatal and nonfatal burden of ACM in China and different regions around the world from 1990 to 2019.

## METHODS

### Study data

Our study was based on 2019 GBD Study [17,18]. All anonymized data were publicly accessible at the Global Health Data Exchange website (http://ghdx.healthda-ta.org/gbd-results-tool) [19]. The general approach for estimating ACM burden from the GBD study has been described and illustrated in detail in the previous study [20,21]. The social-demographic index (SDI) data by 21 GBD regions were obtained from the IHME website (http://ghdx.healthdata.org/). To explore the potential indicators of the burden of ACM in China and globally, we analysed the data in several aspects. First, gender and age-related data were retrieved to assess their impact on the ACM burden. Second, the countries and territories related ACM burden were further categorized by SDI quintiles including high, high-middle, middle, low-middle, and low levels [22]. The data were re-analysed according to 21 GBD regions divided by geographical location. Finally, the burden of ACM in 204 countries and territories was presented by tables and world maps.

### Definition

#### DALYs

Disability-adjusted life years (DALYs) equal the sum of years of life lost (YLLs) and years lived with disability (YLDs). One DALY equals one lost year of healthy life. DALYs allow us to estimate the total number of years lost due to specific causes and risk factors at the country, regional, and global levels.

#### ASDR

The age-standardized death rate (ASDR) is a weighted average of the age-specific mortality rates per 100 000 persons. Age-standardised rate (ASR) is a summary measure of the rate that would have been observed if the population had a standard age structure. Standardisation is necessary when comparing several populations that differ with respect to age, which was calculated by transforming the rate of the practical age composition of population to that of a standard age structure. The weights are derived from the standard population distribution and the ASR (per 100 000 persons) is expressed as:



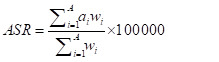



where *a_i_* is the specific age ratio of group *i* and *w_i_* is number or weight of the selected reference standard population group *i*.

#### Age-standardized DALY rate

It is a weighted average of the age-specific DALY rates per 100 000 persons.

#### EAPC

The estimated annual percentage change (EAPC) is a widely used measure to represent the trend of ASDR and age-standardized DALY rate over a certain interval [23]. It is computed by a well-established formula [24], and its 95% uncertainty interval (UI) can also be gotten from regression models. An EAPC of 0, positive or negative value indicates that rates are stable, in a downward or upward trend over time, respectively, and the greater the absolute value of EAPC, the faster the rate changes over time.

#### SDI

It is a summary measure that identifies where countries or other geographic areas sit on the spectrum of development. Expressed on a scale of 0 to 1, it is a composite average of the rankings of the incomes per capita, average educational attainment, and fertility rates of all areas in the GBD study.

### Statistical analysis

We used the deaths, DALYs, ASDR, age-standardized DALY rate, and the corresponding EAPC to assess the ACM burden as well as its variation during the last thirty years. All statistical analyses were performed by R program (Version 4.1.1, R core team) and the *P* < 0.05 were considered as statistically significant.

## RESULTS

### Global burden in 2019

**Figure 1:**
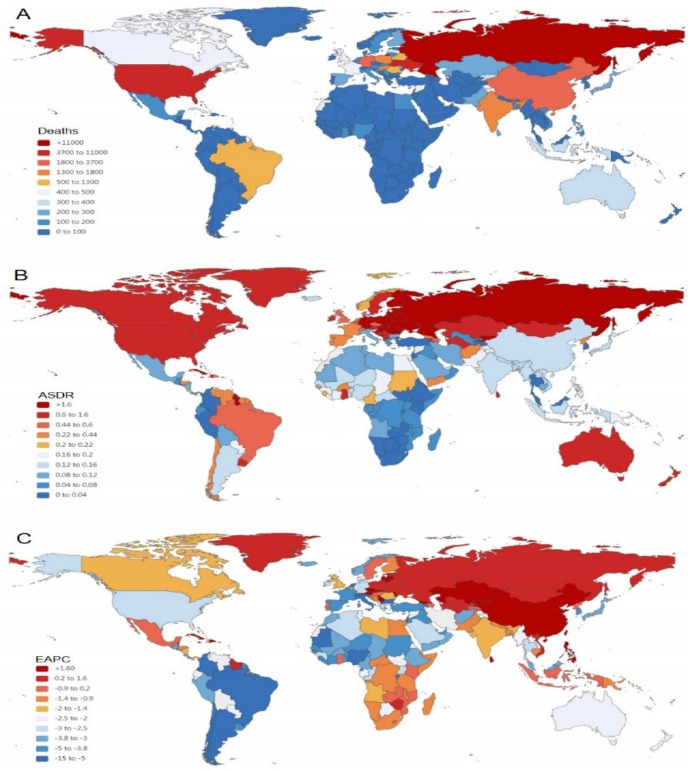
The global deaths burden of ACM in 204 countries and territories. **Panel A.** The absolute number of ACM death cases in 2019; **Panel B.** The ASDR (per 100 000 persons) of ACM in 2019. **Panel C.** The EAPC of ACM ASDRs between 1990 and 2019. ACM – alcoholic cardiomyopathy, ASDR – age standardized death rate, EAPC – estimated annual percentage change.

**Figure 2:**
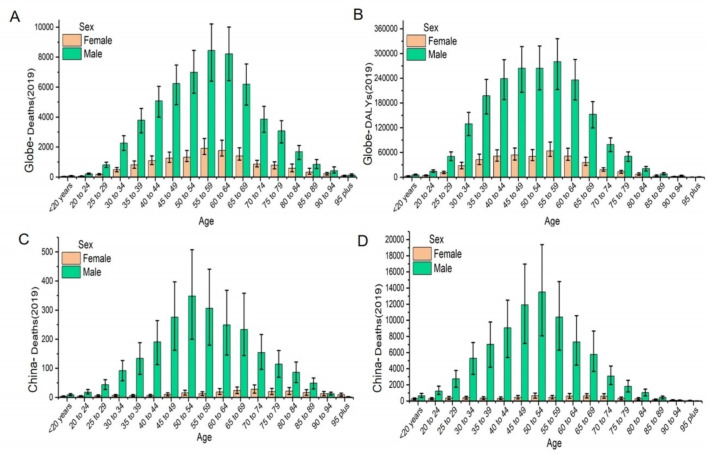
The global ACM burden in both sexes and different age groups in 2019. **Panel A.** The absolute number of global death cases. **Panel B.** The absolute number of global DALYs. **Panel C.** The absolute number of death cases in China. **Panel D.** The absolute number of DALYs in China. ACM – alcoholic cardiomyopathy, DALY – disability-adjusted life year.

Globally, the death cases of ACM were 71 723 with an ASDR of 0.86 per 100 000 persons and the global ACM-related DALYs were 2 441 108 with an age-standardized DALY rate of 29.20 per 100 000 persons in 2019. Among the 21 GBD regions, the highest absolute burden was observed in Eastern Europe (death = 44 024, ASDR = 15.02 per 100 000 persons, DALY = 1 604 440, age-standardized DALY rate = 580.58 per 100 000 persons) (**Table 1**). In 204 countries, the countries with top ranking deaths and DALYs were Russian Federation (death = 31 855, DALY = 1 158 387), Ukraine (death = 10 075, DALY = 37 1847) and United States of America (death = 5826, DALY =  174 249). The highest record of ASDR and age-standardized DALY rate were observed in Latvia (ASDR = 16.31 per 100 000, age-standardized DALY rate =  29.33 per 100 000), Ukraine (ASDR =  16.24 per 100 000, age-standardized DALY rate =  636.34 per 100 000), Russian Federation (ASDR =  15.65 per 100 000, age-standardized DALY rate =  602.19 per 100 000) (Table S1 and S2 in the **Online Supplementary Document**; **Figure 1**, Panel A and Panel B; Figure S1 in the **Online Supplementary Document**, Panel A and Panel B). Men aged 55-59 years old accounted for the majority of the ACM burden (**Figure 2**, Panel A and Panel B).

**Table 1:** The death cases and age-standardized death rate of ACM in 1990 and 2019, and its temporal trends from 1990 to 2019

	1990		2019		1990-2019
**Characteristics**	**Death cases No. ×10^2^ (95% UI)**	**ASDR per 100 000 No. (95% UI)**	**Death cases No. ×10^2^ (95% UI)**	**ASDR per 100 000No. (95% UI)**	**EAPC No. (95% UI)**
**Global**					
Overall	539.02 (495.13-692.48)	1.34 (1.23-1.69)	717.23 (601.66-819.95)	0.86 (0.72-0.99)	-1.52 (-2.39 to -0.65)
Sex:					
Male	372.84 (336.01-513.36)	1.92 (1.74-2.57)	584.39 (469.06-686.67)	1.46 (1.16-1.71)	-1.00 (-1.82 to -0.17)
Female	166.18 (144.84-221.84)	0.8 (0.69-1.04)	132.84 (106.47-171.05)	0.31 (0.25-0.40)	-3.16 (-4.16 to -2.15)
**Social-demographic index:**					
Low	7.68 (4.85-12.00)	0.30 (0.19-0.47)	9.03 (6.55-12.78)	0.16 (0.1-0.23)	-2.43 (-2.56 to -2.30)
Low-middle	19.49 (13.13-28.29)	0.31 (0.2-0.45)	31.52 (24.57-38.43)	0.22 (0.17-0.27)	-1.26 (-1.33 to -1.19)
Middle	24.03 (18.87-32.60)	0.22 (0.17-0.3)	40.33 (30.46-49.70)	0.16 (0.12-0.19)	-0.91 (-1.10 to -0.72)
High-middle	326.40 (280.04-460.94)	3.03 (2.64-4.19)	499.79 (411.89-590.58)	2.58 (2.21-3.03)	-0.62 (-1.85 to 0.62)
High	161.24 (120.61-198.80)	1.60 (1.20-1.96)	136.21 (109.79-165.96)	0.81 (0.65-0.98)	-2.68 (-2.83 to -2.53)
**Regions:**					
Central Asia	3.70 (3.17-4.59)	0.76 (0.64-0.92)	8.13 (4.73-11.55)	0.95 (0.56-1.34)	1.41 (0.61 to 2.21)
Central Europe	37.48 (31.54.-45.85)	2.7 (2.25-3.3)	45.45 (33.23-54.74)	2.37 (1.71-2.86)	-0.16 (-0.26 to -0.06)
Eastern Europe	263.83 (205.24-392.24)	9.97 (7.85-14.4)	440.24 (360.64-526.72)	15.02 (12.29-17.94)	1.41 (-0.06 to 2.91)
Australasia	4.21 (3.28-5.28)	1.84 (1.43-2.3)	4.76 (3.54-6.12)	1.07 (0.8-1.36)	-2.26 (-2.58 to -1.93)
High-income Asia Pacific	4.89 (3.78-5.85)	0.24(0.18-0.28)	3.21 (2.26-4.10)	0.1 (0.07-0.12)	-3.67 (-3.84 to -3.49)
High-income North America	67.36 (48.25-81.16)	2.01(1.45-2.43)	62.33 (50.51-78.31)	1.08 (0.88-1.34	-2.87 (-3.24 to -2.51)
Southern Latin America	5.49 (4.14-7.12)	1.18(0.89-1.53)	1.87 (1.40-2.47)	0.23 (0.17-0.31)	-7.03 (-7.64 to -6.42)
Western Europe	90.80 (69.59-116.89)	1.66(1.26-2.12)	59.70 (47.73-73.05)	0.74 (0.59-0.9)	-3.18 (-3.40 to -2.96)
Andean Latin America	0.23 (0.14-0.34)	0.11(0.07-0.17)	0.27. (0.20-0.38)	0.05 (0.04-0.07)	-2.87 (-3.23 to -2.50)
Caribbean	2.82 (2.18-3.91)	1.04(0.81-1.44)	7.90 (6.26-9.64)	1.52 (1.2-1.85)	2.30 (1.84 to 2.77)
Central Latin America	2.77 (2.04-3.35)	0.31(0.23-0.38)	2.90 (2.20-3.71)	0.12 (0.09-0.15)	-4.05 (-4.41 to -3.69)
Tropical Latin America	17.07 (13.13-21.64)	1.57(1.22-2.01)	12.36 (9.39-15.37)	0.49 (0.37-0.61)	-5.23 (-5.71 to -4.74)
North Africa and Middle East	4.60 (2.44-7.44)	0.27(0.14-0.47)	5.29 (3.49-6.88)	0.12 (0.08-0.16)	-2.98 (-3.04 to -2.93)
South Asia	12.44 (5.68-20.88)	0.22(0.1-0.37)	20.28 (14.30-28.32)	0.14(0.1-0.2)	-1.62 (-1.69 to -1.56)
East Asia	10.43 (5.80-21.08)	0.11(0.06-0.24)	26.80 (18.01-35.71)	0.13(0.09-0.18)	1.58 (0.96 to 2.21)
Oceania	0.09+ (0.05-0.16)	0.29(0.15-0.54)	0.13 (0.07-0.23)	0.18(0.1-0.3)	-1.73 (-1.80 to -1.65)
Southeast Asia	4.26 (2.81-6.74)	0.16(0.11-0.24)	9.46 (6.11-14.76)	0.15(0.09-0.22)	0.28 (0.09 to 0.48)
Central Sub-Saharan Africa	0.32+ (0.17-0.55)	0.12(0.07-0.2)	0.51 (0.27-0.97)	0.08(0.04-0.15)	-1.46 (-1.58 to -1.34)
Eastern Sub-Saharan Africa	0.54 (0.31-1.06)	0.06 (0.03-0.12)	0.76 (0.33-1.34)	0.04(0.02-0.07)	-1.96 (-2.08 to -1.84)
Southern Sub-Saharan Africa	0.12 (0.10-0.16)	0.04 (0.03-0.05)	0.17 (0.13-0.23)	0.03 (0.02-0.04)	-1.04 (-1.28 to -0.80)
Western Sub-Saharan Africa	5.56 (3.39-8.32)	0.55 (0.34-0.83)	4.68 (3.15-6.52)	0.21 (0.14-0.29)	-4.10 (-4.45 to -3.75)
**China:**					
Overall	9.39 (4.94-19.38)	0.11 (0.05-0.23)	25.45 (16.80-34.03)	0.13 (0.09-0.17)	1.97 (1.28 to 2.67)
Sex					
Male	6.13 (3.58-12.32)	0.12 (0.07-0.25)	23.21 (14.43-32.02)	0.24 (0.16-0.34)	3.90(3.07 to 4.74)
Female	3.26 (0.91-8.66)	0.08 (0.02-0.22)	2.23 (1.26-3.29)	0.02 (0.01-0.04)	-3.63 (-3.85 to -3.42)

ACM – alcoholic cardiomyopathy, ASDR – age-standardized death rate, EAPC – estimated annual percentage change

### Relative changes from 1990 to 2019

Globally, the ACM-related deaths and DALYs number in 2019 were 71 723 and 2 441 108, marking a 33.06 and 38.79% increase from 1990, respectively, while the ASDR and age-standardized DALYs rate decreased in 2019 compared to that in 1990, with an overall EAPC of -1.52 (95% UI = -2.39, -0.65) and -1.12 (95% UI = -2.14, -0.10), respectively (**Table 1** and **Table 2**). The most significant ACM burden increase was observed in Eastern Europe, and the most pronounced decrease was in Western Europe, High-income Asia Pacific, Tropical Latin America and High-income North America (**Table 1** and **Table 2**). On observation of ASDR and age-standardized DALY rate from the 204 countries, Republic of Moldova, Latvia, and Kazakhstan were the three countries with the highest EAPC. Slovenia, Italy, and North Macedonia were the three countries with the lowest EAPC (**Figure 1**, Panel C; Figure S1 in the **Online Supplementary Document****,** panel C).

**Table 2:** The DALYs and age-standardized DALYs rate of ACM in 1990 and 2019, and its temporal trends from 1990 to 2019

	1990		2019		1990-2019
**Characteristics**	**DALYs No. ×10^3^ (95% UI)**	**Age-standardized DALY rate per 100 000 No. (95% UI)**	**DALYs No. ×10^3^ (95% UI)**	**Age-standardized DALY rate per 100 000 No. (95% UI)**	**EAPC No. (95% UI)**
**Globe**					
Overall	1758.79 (1594.44-2290.55)	40.22 (36.62-52.06)	2441.11 (2046.73-2782.54)	29.20 (24.51-33.31)	-1.12 (-2.14 to -0.10)
Sex:					
Male	1288.31 (1143.82-1752.45)	59.61 (53.47-80.81)	1999.17 (1626.39-2327.30)	48.59 (39.47-56.58)	-0.75 (-1.72 to 0.22)
Female	470.48 (406.86-674.52)	21.33 (18.49-30.33)	441.94 (354.78-567.05)	10.43 (8.36-13.38)	-2.33 (-3.49 to -1.15)
**Social-demographic index**					
Low	25.62 (16.31-39.65)	8.85 (5.69-13.81)	30.60 (22.63-43.51)	4.76 (3.5-6.79)	-2.44 (-2.57 to -2.30)
Low-middle	66.35 (45.62-93.70)	8.99 (6.14-12.87)	105.70 (81.78-126.45)	6.78 (5.27-8.13)	-1.02 (-1.07 to -0.96)
Middle	85.58 (67.68-115.00)	6.67 (5.27-8.91)	134.26 (103.05-166.36)	5 (3.84-6.18)	-0.80 (-0.98 to -0.63)
High-middle	1118.34 (922.74-1592.21)	98.64 (81.98-139.36)	1780.47 (1474.97-2096.07)	94.53 (78.17-111.01)	-0.25 (-1.58 to 1.11)
High	462.33 (359.51-548.83)	47.59 (37.16-56.34)	389.05 (319.87-459.91)	25.18 (20.67-29.68)	-2.47 (-2.60 to -2.34)
**Region**					
Central Asia	12.66 (10.92-16.44)	24.12 (20.86-31.08)	30.33 (17.70-42.55)	32.59 (19.19-45.74)	1.75 (0.95 to 2.55)
Central Europe	104.901 (88.84-124.78)	73.69 (62.14-87.51)	128.07 (91.50-153.56)	72.56 (51.08-87.16)	0.23 (0.09 to 0.36)
Eastern Europe	936.46 (712.19-1384.38)	356.14 (272.01-518.48)	1604.44 (1318.87-1912.65)	580.58 (476.55-688.97)	1.64 (0.08 to 3.21)
Australasia	13.16 (10.58-16.05)	58.57 (46.93-71.18)	14.17 (10.96-17.48)	34.39 (26.8-42.02)	-2.12 (-2.39 to -1.85)
High-income Asia Pacific	17.46 (14.14-20.67)	8.49 (6.93-10.06)	10.35 (7.66-12.82)	3.64 (2.72-4.5)	-3.44 (-3.60 to -3.27)
High-income North America	204.09 (154.01-239.45)	63.89 (48.3-74.86)	186.69 (156.71-228.77)	34.49 (29.52-42.17)	-2.71 (-3.09 to -2.32)
Southern Latin America	16.65 (12.66-21.34)	35.41 (26.95-45.4)	5.79 (4.43-7.40)	7.41 (5.68-9.47)	-6.78 (-7.34 to -6.21)
Western Europe	237.97 (186.54-292.54)	46.68 (36.52-56.73)	0.69 (0.51-0.93)	21.44 (17.39-25.55)	-3.07 (-3.30 to -2.85)
Andean Latin America	0.68 (0.44-0.95)	2.9 (1.82-4.09)	154.27 (124.67-183.58)	1.34 (0.96-1.83)	-2.71 (-3.05 to -2.36)
Caribbean	9.47 (7.10-13.01)	33.76 (25.61-46.57)	0.80 (0.57-1.09)	45.48 (36.37-55.73)	1.94 (1.52 to 2.35)
Central Latin America	9.03 (6.85-10.47)	8.86 (6.64-10.45)	23.60 (18.89-28.92)	3.78 (2.9-4.78)	-3.48 (-3.82 to -3.14)
Tropical Latin America	64.46 (49.78-80.54)	54.82 (42.37-68.97)	9.54 (7.31-12.03)	17.53 (13.57-21.57)	-5.10 (-5.57 to -4.63)
North Africa and Middle East	14.12 (8.05-21.63)	6.99 (3.82-11.01)	44.75 (34.531-55.14)	3.38 (2.3-4.35)	-2.62 (-2.67 to -2.57)
South Asia	39.53 (17.90-64.52)	5.81 (2.68-9.68)	17.45 (12.04-22.52)	3.91 (2.76-5.47)	-1.47 (-1.54 to -1.40)
East Asia	39.22 (22.97-76.78)	3.64 (2.1-7.14)	60.91 (43.00-84.73)	4.65 (3.15-6.09)	1.88 (1.25 to 2.51)
Oceania	0.31 (0.16-0.55)	8 (4.25-14.14)	92.65 (62.40-122.28)	5.12 (2.73-8.67)	-1.57 (-1.63 to -1.51)
Southeast Asia	15.64 (10.35-24.88)	4.76 (3.21-7.46)	0.47 (0.24-0.79)	4.75 (3.12-7.46)	0.51 (0.31 to 0.72)
Central Sub-Saharan Africa	1.16 (0.61-1.91)	3.85 (2.12-6.49)	33.74 (22.00-54.04)	2.6 (1.43-4.82)	-1.38 (-1.47 to -1.29)
Eastern Sub-Saharan Africa	2.13 (1.30-4.08)	2.15 (1.32-4.08)	1.97 (1.14-3.60)	1.41 (0.69-2.35)	-1.72 (-1.83 to -1.60)
Southern Sub-Saharan Africa	0.51 (0.40-0.66)	1.27 (1-1.67)	3.25 (1.60-5.47)	0.96 (0.73-1.28)	-1.14 (-1.34 to -0.95)
Western Sub-Saharan Africa	19.13 (11.86-28.41)	18.28 (11.36-27.11)	17.19 (11.72-23.75)	6.98 (4.8-9.59)	-3.99 (-4.34 to -3.65)
**China**					
Overall	35.53 (20.07-70.32)	3.41 (1.89-6.89)	87.82 (57.88-117.54)	4.56 (3.01-6.06)	2.18 (1.50 ~ 2.87)
Sex					
Male	24.78 (14.91-48.04)	4.53 (2.73-8.850.25)	81.486 (51.66-111.63)	8.41 (5.34-11.42)	3.60 (2.81 to 4.40)
Female	10.76 (3.35-27.70)	2.15 (0.65-5.68)	6.34 (3.89-111.63)	0.71 (0.45-0.99)	-3.69 (-3.75 to -3.63)

ACM – alcoholic cardiomyopathy, DALY – disability-adjusted life year, EAPC – estimated annual percentage change.

### Disparities across SDI quintiles

**Figure 3:**
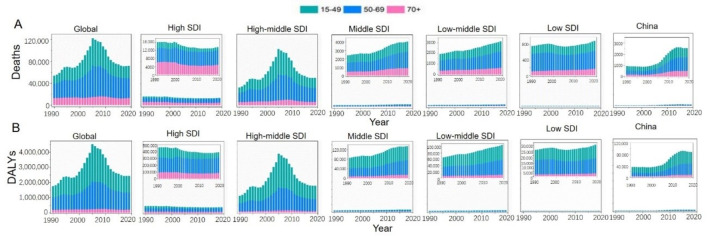
The proportion of the three age groups (15-49 years, 50-69 years and 70+ years) for ACM deaths (**Panel A**) and DALYs (**Panel B**) globally and in five SDI quintiles between 1990 and 2019. ACM – alcoholic cardiomyopathy, SDI – socio-demographic index, DALY – disability-adjusted life year.

**Figure 4:**
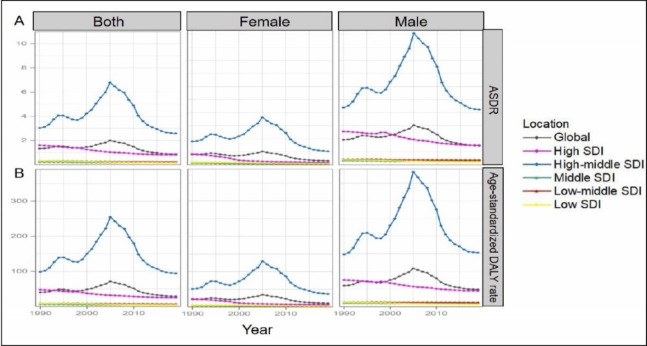
The change trends of ASDR, age-standardized DALY rate globally and among different SDI quintiles between 1990 and 2019. **Panel A.** ASDR; **Panel B.** age standardized DALY rate. SDI – socio-demographic index, ASDR – age-standardized death rate, DALY – disability-adjusted life year.

The global ACM burden varied dramatically among SDI quintiles. In 2019, more than half of the deaths and DALYs were accounted by high-middle SDI quintile (death = 49 979, DALY = 1 780 470). Also, the leading ASDR and age-standardized DALYs rate were observed in high-middle SDI quintile (2.58 and 94.53 per 100 000 respectively). From 1990 to 2019, the high-middle SDI quintile always contributed the most to the ACM burden, which fluctuated significantly and reached the peak in 2005. For deaths and DALYs, the middle and low-middle SDI quintiles showed an upward trend, while the high SDI quintile had the lowest burden and displayed a downward trend (**Figure 3**). For ASDR and age-standardized DALYs rate, the middle, low-middle and low SDI quintiles remained a stable trend (**Figure 4**).

### The relationship between ACM burden and SDI in 21 GBD regions

**Figure 5:**
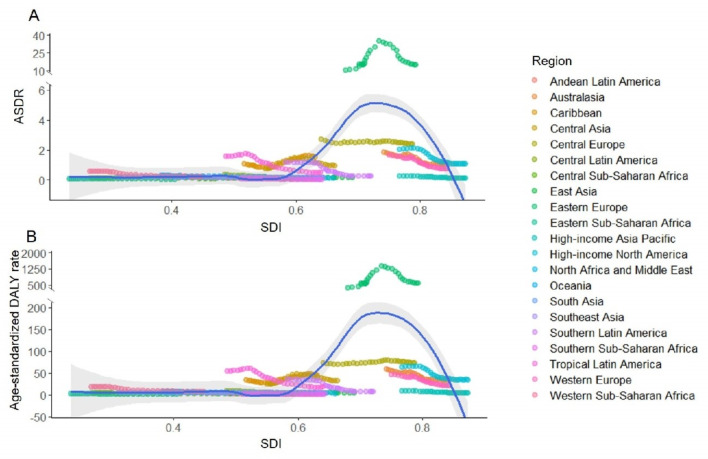
Trends of ASDR and age-standardized DALY rate of ACM in 21 GBD regions from 1990 to 2019. **Panel A.** The ASDR of ACM. **Panel B.** age-standardized DALY rate of ACM. ACM – alcoholic cardiomyopathy, GBD – global burden of diseases, SDI – social-demographic index, ASDR – age-standardized death rate, DALY – disability-adjusted life year.

Taken as a whole, the estimated relationship between SDI and ASDR/age-standardized DALY rate showed an increased trend and stayed at high level for SDI around 0.7, and declined quickly for SDI near 0.8, presenting an asymmetrically inverted U-shaped distribution with the peak value in high-middle SDI regions. Of these, Eastern Europe has consistently had the highest ASDR and age-standardized DALYs rate based on SDI, while most regions displayed a downward trend (**Figure 5**).

### Trends of burden across ages and genders

Globally, the burden of ACM was closely related to gender and age. Typical sex difference existed in ACM-related deaths and DALYs. Males had much higher death numbers and DALYs than females, and even more than four-fifths of the deaths and DALYs were recorded for males. Overall, the annual absolute value of ASDR and age-standardized DALYs rate for males outnumbered that for females globally and in all SDI quintiles. Some other notable patterns were observed in the high-middle SDI region for males, where the ASDR and age-standardized DALYs rate increased consistently on a higher base, peaking in 2005, and then subsequently experiencing a faster decline than that for females (**Figure 4**).

The number of deaths and DALYs were highest for the age group of 50 to 69 for both of male and female, while the ones for the age group of 70+ remained few and stable from 1990 to 2019 (**Figure 3**). In Eastern Europe, the 50-69 years-group accounted for most of the DALY number in 1990, while in 2019, the 15-49 years group experienced a sharp increase compared to 1990 (Figure S1 in the **Online Supplementary Document**).

### ACM burden in China

**Figure 6:**
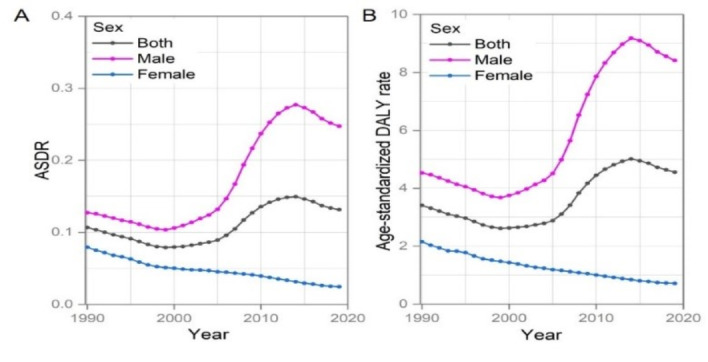
The change trends of ASDR, age-standardized DALY rate in China between 1990 and 2019. **Panel A.** ASDR. **Panel B.** age standardized DALY rate. ASDR – age-standardized death rate, DALY – disability-adjusted life year.

In 2019, with 2545 deaths related to ACM, China was the fifth country with the highest death number after the Russian Federation, Ukraine, the United States and Germany (Table S1A and B in the **Online Supplementary Document**). The ACM DALYs in China were 87 823, and it was the fourth country with the largest number of DALYs after the Russian Federation, Ukraine, United States of America (Table S1 in the **Online Supplementary Document**, Figure S1 in the **Online Supplementary Document**, Panel A and panel B). From 1990 to 2005, deaths and DALYs remained stable, then increased sharply, peaking in 2014. The predominant age composition gradually changed from the 15-19 age group to the 50-69 age group over the past three decades (**Figure 3**). The ASDR and age-standardized DALY rate of ACM was 0.13 per 100 000 and 4.56 per 100 000 in 2019, respectively. They fluctuated considerably over the past three decades. ASDR for men has remained at a higher level than that for women, which increased rapidly from 2000 to 2014 and then gradually declined. At the same time, ASDR for women has maintained a moderate downward trend. Age-standardized DALY rate has remained a similar trend to ASDR. Overall, the ACM burden was much higher for men than for women (**Figure 6**).

## DISCUSSION

Globally, the burden of ACM has been increasing, both fatal and nonfatal. The global deaths and DALYs number increased dramatically from 1990 to 2019, while the global ASDR and age-standardized DALYs rate decreased, indicating that population ageing and growth mostly accounted for the absolute increase in the number of ACM cases. Substantive discrepancies existed in ACM burden across SDI quintiles. The largest absolute burden and most pronounced ASDR increases and age-standardized DALYs rate were observed in the high-middle SDI quintile, while the middle, low-middle and low SDI quintiles had the lowest burdens. The high SDI quintile showed a slightly downward trend for death, DALYs number and ASDR/age-standardized DALY rate. Middle-aged men had largely outpaced women in absolute fatal and nonfatal burden, with a much higher ASDR and age-standardized DALYs rate than that of female. Overall, these results demonstrated the importance of improving strategies in prevention and treatment of ACM for middle-aged adults across the world, especially for men in high-middle SDI countries.

Globally, Eastern Europe has been identified as the region with the highest levels of alcohol-related health damage and mortality [25-27]. More than 90% of countries in the region consumed more than two litres of anhydrous alcohol per capita per year [28]. Despite the high burden of alcohol harm and mortality in Eastern Europe, very few policies had been implemented. Moreover, most countries did not advertise the dangers of excessive alcohol consumption and related laws and regulations on TV, radio or the Internet. Although an international symposium on “The Ongoing Alcohol Burden in Central and Eastern Europe” was held in June 2017 to outline the issue of alcohol-related mortality in a range of Eastern European countries and linked it to alcohol control initiatives, the sustainability of alcohol restriction policies and measures was of concern due to persistent socio-political, social divisions and highly volatile election outcomes in Eastern Europe [29-31].

The relationship between SDI and ASDR/age-standardized DALYs rate indicated that the high-middle SDI countries were bearing the heavy and increasing burden, and this situation was likely the past of the high SDI regions but the near future of the middle SDI regions. It will be crucial for government agencies to enact strong alcohol control policies in the middle SDI regions and maintain that in the high SDI regions in the future. In the high-middle SDI countries such as Belarus, Argentina, Kazakhstan, Portugal and Spain, the heavy burden of ACM may be related to absolute excessive alcohol consumption. It is urgent to require more targeted strategies to reduce alcohol consumption. However, the low SDI countries such as Pakistan, Haiti, Afghanistan and Ethiopia also had the high burden of ACM, which may be associated with low economic development and poorer medical conditions, so increased investment and improved medical services in prevention and treatment of ACM are important controlling the burden.

Our data showed that sex preponderance existed undoubtedly in ACM burden. First, men consumed more alcohol than women [32-34], and male drinkers consumed larger quantities of alcohol than female drinkers [35-37]. This may be due to the fact that men's social roles and occupations made them more likely and more frequently exposed to alcohol. Second, our results were consistent to the study from Nationwide Inpatient Sample databases. The study demonstrated that the ratio of male to female prevalence of ACM was 8:1 and men with ACM had a higher risk of developing heart failure compared to women [38].

China had a long history of alcohol production and consumption. Especially, excessive alcohol consumption on special occasions such as rituals, funerals, marriages, and festivals has been a custom for thousands of years. In recent decades, the consumption of alcohol in China has grown rapidly compared to other countries, which is closely related to the trend of socioeconomic development and increased alcohol production in China [39]. Statistics from the World Health Organization show that the per capita consumption of pure alcohol in China was 1.03 L in 1970 and had risen to 5.17 L in 1996 and 5.91 L in 2011 [14]. In terms of taxation, the State Council adopted the Provisional Regulations of the People's Republic of China, which expanded the tax on alcohol from a simple production tax to a consumption tax at a reduced rate compared to the original levy in 1994. The government discontinued differential taxation on potatoes and grain alcohol, driving a dramatic increase in alcohol production in 2006 [39]. This may explain the gradual increase in the burden of ACM after 2005 in China.

Alcohol consumption is closely related to public health [40]. ACM are highly preventable, and timely intervention significantly improves outcomes. Our study improved the understanding about levels and trends of the ACM in China and different regions around the world, and hoped to appropriately guide efforts of improving cardiovascular health in early stage at macro geographical scale. The Chinese government should revise the current industrial policies that encourage and support the development of the alcohol industry by strengthening taxation, regulating the production of various types of alcoholic beverages, reducing alcohol concentration, strengthening retail management [41], preventing illegal alcoholic beverages and reducing informal alcohol, and revising the Alcohol Advertising Regulations to regulate advertising, sponsorship and promotional activities and restrict new media such as the Internet [42].

## CONCLUSIONS

In summary, the burden of ACM has considerable spatial and temporal heterogeneity and gender heterogeneity around the world. The total burden is highest in high-middle SDI regions, especially in Eastern Europe, and the burden in men largely outweigh women. The burden in China has increased rapidly in the last three decades. Our findings may provide policy makers with some references for targeted strategic preventive measures that should be taken in priority regions and countries.

## Additional material


Online Supplementary Document
