# Supplementary material for: The burden of alcoholic cardiomyopathy in China and different regions around the world
Source: J Glob Health. 2022 Jul 30;12:04041. doi: 10.7189/jogh.12.04041 (PMC9304924; doi:10.7189/jogh.12.04041)
Supplement: Online Supplementary Document [file jogh-12-04041-s001.pdf]

The burden of alcoholic cardiomyopathy in China and different regions around the world

Jing Zhang [1, 2, 3#], Hailing Liu [4#], Xiang Bu [5], Qun Lu [1, 2, 3], Lu Cheng[6], Aiqun Ma [1, 2, 3*], Tingzhong Wang [1, 2, 3*]

[1] Department of Cardiovascular Medicine, The First Affiliated Hospital of Xi'an Jiaotong University, Xi'an, Shaanxi, China

[2] Key Laboratory of Molecular Cardiology, Xi'an, Shaanxi, China

[3] Key Laboratory of Environment and Genes Related to Diseases, Xi'an Jiaotong University, Ministry of Education, China

[4] Department of Pediatrics, The First Affiliated Hospital of Xi'an Jiaotong University, Xi'an, Shaanxi, China

[5] Department of Respiratory and Critical Care Medicine, The First Affiliated Hospital of Xi'an Jiaotong University, Xi'an, Shaanxi, China

[6] Department of Cardiovascular Medicine, The Affiliated Cardiovascular Hospital of Qingdao University, Qingdao, Shandong, China

[#]These authors have contributed equally to this work.

*Corresponding authors at: Department of Cardiovascular Medicine, First Affiliated Hospital of Xi'an Jiaotong University, No. 277 Yanta West Road, Xi'an 710061, Shaanxi, China.

E-mail addresses: aiqun.ma@xjtu.edu.cn (A. Ma), tingzhong.wang@xjtu.edu.cn (T. Wang)

Table S1. The death cases and age-standardized death rate of ACM in 1990 and 2019, and its temporal trends in 204 countries and territories from 1990 to 2019.

	1990		2019		1990-2019
Countries and territories	Death cases No.(95%UI)	ASDR per 100,000 No. (95%UI)	Death cases No. (95%UI)	ASDR per 100,000No. (95%UI)	EAPC No. (95%UI)
Afghanistan	54.37(12.55-114.3)	0.79(0.17-1.78)	49.35(14.05-88.77)	0.39(0.1-0.8)	-3.00 (-3.52~-2.48)
Albania	27.28(19.77-36.21)	1.4(0.98-1.8)	29.42(17.55-51.68)	0.73(0.44-1.29)	-2.04 (-2.35~-1.74)
Algeria	25.4(14.55-40.54)	0.24(0.13-0.39)	33.91(15.48-55.36)	0.11(0.05-0.18)	-2.80 (-2.93~-2.66)
American Samoa	0.14(0.09-0.2)	0.58(0.37-0.8)	0.07(0.05-0.11)	0.15(0.11-0.22)	-5.58 (-6.06~-5.10)
Andorra	1.07(0.66-1.68)	1.94(1.19-3.02)	1.54(1.06-2.09)	1.09(0.76-1.49)	-2.24 (-2.37~-2.10)
Angola	6.16(2.79-11.15)	0.13(0.06-0.23)	11.86(6.9-19.31)	0.08(0.05-0.14)	-1.55 (-1.64~-1.46)
Antigua and Barbuda	0.13(0.1-0.19)	0.26(0.19-0.38)	0.52(0.3-0.71)	0.49(0.28-0.66)	3.34 (2.67~4.01)
Argentina	354.16(252.12-478.5)	1.09(0.78-1.46)	81.06(60.68-107.8)	0.16(0.12-0.21)	-8.49 (-9.35~-7.62)
Armenia	127.53(96.1-158.37)	5.31(3.6-6.84)	52.62(34.9-86.07)	1.37(0.92-2.19)	-5.41 (-6.54~-4.28)
Australia	340.55(271.08-420.46)	1.78(1.42-2.19)	391.3(282.58-521)	1.04(0.75-1.38)	-2.24 (-2.59~-1.89)
Austria	35.22(24.56-105.39)	0.3(0.22-0.86)	126.57(88.81-174.69)	0.75(0.51-1.05)	2.04 (0.59~3.52)
Azerbaijan	28.12(12.67-66.6)	0.52(0.23-1.22)	48.03(21.65-136.63)	0.47(0.22-1.24)	-0.88 (-1.25~-0.52)
Bahamas	2.96(2.23-4.14)	1.71(1.27-2.39)	8.79(6.37-11.66)	2.05(1.5-2.73)	1.69 (1.15~2.24)
Bahrain	0.65(0.44-1.11)	0.45(0.29-0.84)	1.7(0.95-2.78)	0.26(0.16-0.39)	-2.02 (-2.55~-1.49)
Bangladesh	103.59(40.45-176.66)	0.21(0.09-0.36)	185.39(91.84-304.08)	0.14(0.07-0.23)	-1.20 (-1.36~-1.04)
Barbados	3.59(2.76-4.82)	1.39(1.07-1.83)	8.26(5.92-10.57)	1.76(1.26-2.25)	1.30 (1.01~1.59)
Belarus	755.15(525.49-1033.58)	6.12(4.25-8.39)	1127.68(768.23-1592.43)	8.36(5.69-11.45)	1.01 (0.63~1.38)
Belgium	118.88(86.9-146.35)	0.8(0.59-0.97)	113.16(82-149.81)	0.56(0.39-0.74)	-1.77 (-2.31~-1.22)
Belize	0.29(0.16-0.46)	0.29(0.17-0.46)	1.6(1.05-2.04)	0.52(0.34-0.66)	3.21 (2.75~3.67)
Benin	8.28(4.52-13.51)	0.39(0.21-0.65)	8.19(4.67-14.26)	0.15(0.08-0.25)	-4.03 (-4.41~-3.66)
Bermuda	0.21(0.13-0.4)	0.33(0.2-0.63)	0.45(0.27-0.6)	0.37(0.22-0.5)	1.28 (0.56~2.00)
Bhutan	0.49(0.18-0.96)	0.2(0.07-0.41)	0.78(0.48-1.27)	0.14(0.08-0.22)	-1.41 (-1.49~-1.33)
Bolivia	6.87(3.07-12.78)	0.22(0.1-0.44)	8.74(5.21-12.97)	0.1(0.06-0.15)	-2.57 (-2.90~-2.24)
Bosnia and Herzegovina	74.89(38.04-119.04)	2.09(1.05-3.44)	86.23(39.94-145.95)	1.57(0.72-2.65)	-0.98 (-1.28~-0.69)
Botswana	0.35(0.23-0.54)	0.06(0.04-0.08)	0.56(0.34-0.87)	0.04(0.02-0.05)	-2.19 (-2.60~-1.77)
Brazil	1700.2(1308.55-2156.88)	1.6(1.24-2.05)	1226.57(928.74-1528.74)	0.5(0.38-0.62)	-5.24 (-5.72~-4.76)
Brunei Darussalam	3.24(2.19-4.8)	2.45(1.64-3.58)	2.89(2.1-4.12)	0.84(0.61-1.18)	-3.94 (-4.24~-3.64)
Bulgaria	23.57(13.24-35.23)	0.21(0.12-0.31)	12.31(8.26-21.33)	0.1(0.07-0.17)	-5.88 (-8.11~-3.60)
Burkina Faso	27.29(15.15-48.29)	0.56(0.31-0.98)	24.44(13.78-43.25)	0.24(0.13-0.41)	-3.50 (-3.85~-3.14)
Burundi	4.23(1.7-9.69)	0.15(0.06-0.36)	3.18(1.38-6.42)	0.05(0.02-0.11)	-4.51 (-4.93~-4.09)
Cabo Verde	0.15(0.1-0.23)	0.06(0.04-0.1)	0.36(0.23-0.54)	0.08(0.05-0.11)	-0.34 (-0.69~0.00)
Cambodia	6.89(2.98-14.89)	0.14(0.07-0.31)	13.61(8-27.25)	0.11(0.07-0.22)	-0.92 (-1.05~-0.78)
Cameroon	23.2(12.26-40.49)	0.44(0.23-0.76)	29.62(17.03-49.78)	0.2(0.12-0.34)	-3.13 (-3.38~-2.89)
Canada	283.41(226.16-339.47)	0.89(0.71-1.06)	406.34(294.6-512.23)	0.64(0.47-0.81)	-1.56 (-1.81~-1.31)
Central African Republic	2.7(1.15-5.11)	0.2(0.09-0.37)	3.65(1.58-7.43)	0.14(0.07-0.28)	-1.34 (-1.44~-1.24)
Chad	12.26(6.23-22.95)	0.41(0.21-0.77)	12.27(6.2-22.22)	0.19(0.1-0.34)	-3.23 (-3.54~-2.92)
Chile	106.31(80.21-129.84)	1.01(0.76-1.24)	55.91(40.05-73.19)	0.24(0.17-0.31)	-5.44 (-5.98~-4.90)
China	938.93(493.81-1937.58)	0.11(0.05-0.23)	2544.61(1679.58-3402.55)	0.13(0.09-0.17)	1.97 (1.28~2.67)
Colombia	73.41(51.45-84.3)	0.39(0.27-0.45)	16.63(10.9-26.69)	0.03(0.02-0.05)	-9.19 (-10.76~-7.59)

Comoros	0.11(0.04-0.22)	0.04(0.02-0.09)	0.14(0.04-0.28)	0.03(0.01-0.05)	-2.24 (-2.62~-1.86)
Congo	1.8(0.79-3.18)	0.14(0.07-0.25)	2.27(1.27-3.84)	0.07(0.04-0.12)	-2.73 (-2.97~-2.49)
Cook Islands	0.01(0.01-0.02)	0.1(0.06-0.14)	0.01(0.01-0.01)	0.04(0.03-0.06)	-3.51 (-3.85~-3.16)
Costa Rica	7.01(5.15-9.14)	0.36(0.26-0.47)	10.65(7.06-15.13)	0.2(0.13-0.29)	-3.01 (-3.86~-2.16)
Croatia	83.75(68.39-116.1)	1.56(1.26-2.07)	219.02(156.05-293.31)	2.61(1.87-3.49)	-0.93 (-3.01~1.20)
Cuba	91.85(75.67-132.93)	0.89(0.73-1.29)	488.79(358.71-616.81)	2.67(1.95-3.36)	5.58 (4.90~6.26)
Cyprus	4.39(2.73-6.54)	0.52(0.31-0.79)	4.17(2.94-6.03)	0.22(0.15-0.31)	-3.43 (-3.73~-3.13)
Czechia	12.48(9.37-24.7)	0.1(0.07-0.19)	89.25(53.1-121.47)	0.54(0.31-0.73)	6.45 (5.63~7.27)
C 么 te d'Ivoire	28.18(14.96-47.09)	0.56(0.29-0.93)	24.69(14.39-39.56)	0.19(0.11-0.3)	-4.67 (-5.11~-4.23)
Democratic People's Republic of Korea	37.13(18.23-76.3)	0.22(0.11-0.46)	69.08(42.98-123.1)	0.22(0.14-0.4)	0.16 (0.02~0.29)
Democratic Republic of the Congo	20.12(10.86-35.96)	0.11(0.06-0.2)	32.39(13.63-68.05)	0.08(0.03-0.16)	-1.28 (-1.46~-1.09)
Denmark	52.99(40.47-68.49)	0.76(0.57-0.97)	44.01(32.3-54.6)	0.45(0.33-0.55)	-2.35 (-2.97~-1.73)
Djibouti	0.09(0.03-0.18)	0.04(0.02-0.09)	0.25(0.08-0.52)	0.03(0.01-0.06)	-1.32 (-1.41~-1.22)
Dominica	0.38(0.26-0.57)	0.58(0.4-0.89)	0.74(0.46-1.05)	0.84(0.52-1.19)	2.03 (1.52~2.55)
Dominican Republic	9.72(6.55-14.81)	0.23(0.15-0.35)	44.55(24.66-66.18)	0.45(0.25-0.67)	3.33 (3.04~3.62)
Ecuador	4.31(2.91-6.64)	0.08(0.05-0.12)	6.09(4.21-8.62)	0.04(0.03-0.06)	-2.00 (-2.62~-1.38)
Egypt	86.34(42.76-143.83)	0.3(0.14-0.53)	118.02(57.36-212.1)	0.18(0.08-0.32)	-1.38 (-1.56~-1.19)
El Salvador	1.78(1.13-2.44)	0.06(0.04-0.08)	1.51(0.97-2.13)	0.02(0.02-0.03)	-3.85 (-4.32~-3.38)
Equatorial Guinea	0.4(0.16-0.77)	0.18(0.08-0.33)	0.34(0.17-0.64)	0.05(0.03-0.1)	-4.73 (-5.39~-4.06)
Eritrea	0.73(0.3-1.69)	0.05(0.02-0.13)	1.14(0.53-2.24)	0.03(0.01-0.06)	-2.13 (-2.30~-1.96)
Estonia	77.65(54.18-108.5)	3.95(2.76-5.46)	91.4(61.45-123.39)	4.37(2.99-5.89)	0.65 (-0.35~1.66)
Eswatini	0.17(0.11-0.25)	0.05(0.03-0.08)	0.24(0.14-0.4)	0.04(0.02-0.06)	-0.99 (-1.30~-0.67)
Ethiopia	11.89(4.45-30.88)	0.05(0.02-0.13)	10.92(3.68-21.34)	0.02(0.01-0.04)	-3.27 (-3.41~-3.12)
Fiji	0.24(0.17-0.34)	0.05(0.04-0.08)	0.28(0.18-0.39)	0.04(0.02-0.05)	-1.79 (-2.06~-1.52)
Finland	239.12(170.96-302.35)	3.67(2.58-4.65)	230.15(175.57-269.62)	2.45(1.78-2.82)	-1.07 (-1.45~-0.68)
France	897.42(685.8-1077.19)	1.14(0.89-1.36)	486.52(322.32-689.95)	0.43(0.28-0.6)	-4.06 (-4.75~-3.37)
Gabon	0.87(0.38-1.71)	0.14(0.06-0.28)	0.88(0.5-1.43)	0.07(0.04-0.11)	-2.38 (-2.48~-2.27)
Gambia	1.71(0.85-2.95)	0.43(0.22-0.74)	1.88(1.08-3.13)	0.18(0.1-0.3)	-3.73 (-4.09~-3.36)
Georgia	9.32(4.69-13.85)	0.16(0.08-0.24)	11.58(7.25-23.58)	0.24(0.15-0.47)	1.83 (1.48~2.19)
Germany	5160.51(3823.65-6574.94)	4.27(3.14-5.41)	3602.84(2873.83-4298.01)	2.05(1.64-2.41)	-2.55 (-2.85~-2.25)
Ghana	50.01(30.53-77.06)	0.67(0.41-1.05)	123.39(71-189.99)	0.64(0.36-1)	0.01 (-0.23~0.25)
Greece	16.11(11.19-24.95)	0.12(0.08-0.18)	9.9(7.36-16.99)	0.05(0.04-0.1)	-2.60 (-2.84~-2.35)
Greenland	0.63(0.43-0.9)	1.39(0.94-2)	0.9(0.55-1.28)	1.2(0.73-1.66)	0.24 (-0.34~0.82)
Grenada	0.98(0.7-1.44)	1.5(1.08-2.21)	2.36(1.45-2.96)	1.98(1.19-2.48)	1.84 (1.41~2.27)
Guam	0.48(0.33-0.65)	0.62(0.42-0.85)	0.22(0.14-0.37)	0.11(0.08-0.19)	-6.78 (-7.17~-6.39)
Guatemala	5.03(3.07-7.71)	0.15(0.09-0.23)	6.64(3.94-9.01)	0.06(0.03-0.08)	-3.58 (-4.18~-2.98)
Guinea	12.78(5.96-24.11)	0.36(0.17-0.67)	10.37(5.67-18.14)	0.17(0.09-0.29)	-2.78 (-3.01~-2.54)
Guinea-Bissau	3.01(1.12-6.41)	0.66(0.25-1.4)	1.96(1.08-3.49)	0.23(0.12-0.41)	-4.07 (-4.35~-3.79)
Guyana	13.02(10.21-16.17)	3(2.36-3.73)	15.43(9.69-21.43)	2.21(1.39-3.06)	0.34 (-0.74~1.43)
Haiti	96.9(41.19-165.82)	2.64(1.13-4.64)	128.93(66.41-223.83)	1.62(0.86-2.82)	-1.42 (-1.69~-1.15)
Honduras	12.51(6.1-18.51)	0.57(0.28-0.85)	25.94(12.48-37.83)	0.43(0.21-0.62)	-0.99 (-1.20~-0.78)
Hungary	878.05(756.84-1142.93)	6.52(5.61-8.4)	1311.32(957.74-1688.47)	7.62(5.47-9.82)	0.09 (-0.38~0.56)
Iceland	0.9(0.69-1.15)	0.32(0.25-0.41)	0.75(0.58-0.95)	0.14(0.11-0.18)	-3.16 (-3.32~-3.00)
India	997.69(457.77-1666.01)	0.22(0.1-0.38)	1604.25(1110.02-2251.18)	0.14(0.1-0.19)	-1.74 (-1.83~-1.66)

Indonesia	168.19(94.09-273.44)	0.16(0.09-0.27)	312.23(170.09-584.16)	0.14(0.08-0.25)	-0.02 (-0.15~0.12)
Iran (Islamic Republic of)	54.97(39.31-84.57)	0.21(0.15-0.31)	87.62(53.81-117.21)	0.12(0.07-0.16)	-1.76 (-2.09~-1.43)
Iraq	10.44(5.34-20.28)	0.14(0.07-0.29)	10.3(7.12-15.25)	0.05(0.03-0.07)	-4.81 (-5.26~-4.36)
Ireland	46.32(38.44-63.65)	1.18(0.99-1.62)	49.13(32.23-67.85)	0.69(0.46-0.95)	-2.85 (-3.37~-2.33)
Israel	9.61(6.19-12.49)	0.2(0.13-0.27)	4.79(2.88-6.63)	0.05(0.03-0.06)	-6.82 (-8.52~-5.08)
Italy	796.62(577.88-1545.27)	0.95(0.7-1.79)	103.18(67.99-180.16)	0.09(0.05-0.16)	-11.17 (-12.53~-9.79)
Jamaica	3.86(2.61-6.12)	0.23(0.15-0.37)	15.21(10.48-21.83)	0.51(0.35-0.74)	4.06 (3.36~4.77)
Japan	439.1(324.31-524.04)	0.26(0.2-0.32)	287.3(194.19-375.89)	0.12(0.08-0.16)	-3.12 (-3.28~-2.95)
Jordan	1.23(0.71-1.83)	0.09(0.05-0.14)	2.3(1.64-3.17)	0.03(0.02-0.04)	-4.60 (-5.00~-4.20)
Kazakhstan	30.52(11.3-111.14)	0.22(0.08-0.8)	207.93(75.15-393.99)	1.08(0.38-2.04)	8.12 (5.85~10.44)
Kenya	3.06(1.28-5.8)	0.03(0.01-0.06)	7.65(3.38-13.21)	0.03(0.01-0.05)	-0.41 (-0.55~-0.26)
Kiribati	0.12(0.07-0.18)	0.3(0.17-0.43)	0.18(0.09-0.28)	0.23(0.11-0.35)	-0.63 (-0.93~-0.32)
Kuwait	0.63(0.5-0.86)	0.08(0.06-0.12)	1.32(0.89-1.75)	0.04(0.03-0.06)	-2.32 (-3.12~-1.51)
Kyrgyzstan	134.52(94.51-157.46)	4.11(2.91-4.81)	372.58(182.44-465.49)	6.5(3.29-8.1)	2.44 (1.85~3.03)
Lao People's Democratic Republic	5.95(2.01-12.17)	0.28(0.1-0.56)	7.76(4.46-13.72)	0.16(0.1-0.26)	-2.04 (-2.13~-1.95)
Latvia	98.36(73.81-151.13)	2.98(2.26-4.54)	449.82(300.09-585.4)	16.31(10.95-21.08)	8.38 (6.82~9.96)
Lebanon	5.93(3.47-8.81)	0.27(0.16-0.39)	6.35(2.48-10.24)	0.12(0.05-0.2)	-2.75 (-2.87~-2.64)
Lesotho	0.51(0.28-0.87)	0.05(0.03-0.09)	0.59(0.36-0.97)	0.05(0.03-0.07)	0.18 (-0.03~0.40)
Liberia	5.94(3.17-10.06)	0.51(0.27-0.85)	3.97(1.7-7.92)	0.16(0.07-0.32)	-4.69 (-5.15~-4.24)
Libya	3.08(1.56-5.2)	0.15(0.07-0.26)	5.35(2.49-8.36)	0.1(0.04-0.15)	-1.63 (-1.86~-1.41)
Lithuania	135.86(109.11-174.17)	3.17(2.54-4.05)	195.66(141.57-257.71)	4.74(3.48-6.23)	2.19 (0.55~3.85)
Luxembourg	6.74(5.07-8.21)	1.27(0.95-1.55)	4.84(3.05-6.59)	0.5(0.31-0.68)	-3.71 (-3.88~-3.54)
Madagascar	4.65(2.34-8.43)	0.07(0.04-0.13)	8.21(3.19-15.13)	0.05(0.02-0.1)	-1.25 (-1.34~-1.15)
Malawi	2.7(1.33-5.21)	0.06(0.03-0.12)	4.15(1.54-7.82)	0.05(0.02-0.09)	-1.14 (-1.30~-0.97)
Malaysia	6.79(4.83-10.14)	0.07(0.05-0.1)	8.15(5.4-11.83)	0.03(0.02-0.04)	-3.75 (-4.07~-3.42)
Maldives	0.12(0.05-0.22)	0.12(0.04-0.21)	0.13(0.07-0.19)	0.03(0.02-0.05)	-4.80 (-5.18~-4.42)
Mali	14.43(7.91-27.29)	0.31(0.17-0.58)	11.98(6.01-21.31)	0.12(0.06-0.22)	-3.66 (-4.08~-3.23)
Malta	1.81(1.4-2.26)	0.43(0.33-0.53)	1.64(1.06-2.19)	0.19(0.12-0.25)	-3.00 (-3.15~-2.84)
Marshall Islands	0.07(0.03-0.12)	0.43(0.21-0.79)	0.09(0.04-0.14)	0.25(0.13-0.4)	-1.99 (-2.05~-1.93)
Mauritania	4.34(2.26-7.75)	0.4(0.21-0.7)	2.32(0.83-4.45)	0.1(0.04-0.19)	-5.00 (-5.36~-4.64)
Mauritius	3.42(2.41-4.75)	0.37(0.27-0.52)	3.29(2.3-4.61)	0.21(0.15-0.29)	-2.97 (-4.07~-1.86)
Mexico	74.99(60.66-96.79)	0.16(0.13-0.21)	138.75(103.57-187.18)	0.11(0.09-0.15)	-0.85 (-1.21~-0.49)
Micronesia (Federated States of)	0.2(0.08-0.39)	0.42(0.19-0.87)	0.17(0.07-0.32)	0.23(0.11-0.4)	-2.22 (-2.47~-1.96)
Monaco	0.52(0.33-0.75)	0.85(0.55-1.2)	0.36(0.24-0.5)	0.43(0.29-0.6)	-2.53 (-2.71~-2.34)
Mongolia	8.69(4.58-17.29)	0.7(0.37-1.38)	36.72(21.51-69.17)	1.01(0.6-1.89)	1.35 (1.22~1.47)
Montenegro	78.29(32.56-131.52)	13.38(5.28-23.04)	86.37(42.97-133.11)	9.15(4.61-14.02)	-1.42 (-1.70~-1.15)
Morocco	54.8(24.05-108.64)	0.42(0.17-0.89)	56.07(30.41-82.69)	0.19(0.1-0.27)	-3.04 (-3.17~-2.91)
Mozambique	4.24(1.46-9.25)	0.06(0.02-0.13)	7.77(3.6-16.49)	0.06(0.02-0.12)	-0.06 (-0.19~0.08)
Myanmar	56.44(23.9-108.38)	0.23(0.1-0.43)	58.34(36.92-99.49)	0.12(0.08-0.2)	-2.34 (-2.44~-2.25)
Namibia	0.35(0.21-0.55)	0.05(0.03-0.08)	0.54(0.36-0.85)	0.04(0.02-0.06)	-1.26 (-1.46~-1.06)
Nauru	0.02(0.01-0.03)	0.34(0.17-0.64)	0.01(0.01-0.02)	0.22(0.11-0.42)	-1.42 (-1.79~-1.05)
Nepal	19.88(9.92-34.44)	0.21(0.1-0.38)	34.43(19.3-56.8)	0.16(0.09-0.26)	-1.04 (-1.17~-0.92)
Netherlands	405.27(307.13-500.6)	2.07(1.57-2.55)	157.49(95.43-240.51)	0.5(0.3-0.75)	-5.82 (-6.20~-5.43)
New Zealand	80.3(56.39-109.6)	2.13(1.48-2.91)	84.31(56.37-99.38)	1.2(0.81-1.4)	-2.32 (-2.70~-1.94)

Nicaragua	2.19(1.51-2.97)	0.13(0.09-0.18)	3.33(2.31-4.41)	0.08(0.05-0.11)	-1.98 (-2.53~-1.43)
Niger	13.59(6.96-26.33)	0.42(0.21-0.82)	13.5(6.21-25.78)	0.15(0.07-0.28)	-4.04 (-4.38~-3.71)
Nigeria	320.27(166.55-536.66)	0.63(0.33-1.06)	171.33(95.95-274.83)	0.16(0.09-0.25)	-5.82 (-6.33~-5.30)
Niue	0.01(0-0.01)	0.23(0.15-0.35)	0(00)	0.13(0.08-0.2)	-2.28 (-2.45~-2.12)
North Macedonia	73.27(50.24-101.18)	4.97(3.21-7.01)	16.11(8.73-28.21)	0.6(0.35-1.07)	-9.69 (-10.95~-8.41)
Northern Mariana Islands	0.19(0.12-0.28)	0.94(0.62-1.32)	0.12(0.08-0.19)	0.2(0.14-0.34)	-5.97 (-6.49~-5.45)
Norway	32.58(25.45-38.36)	0.59(0.46-0.68)	16.84(12.88-23.98)	0.21(0.16-0.29)	-3.49 (-3.89~-3.09)
Oman	1.26(0.6-2.33)	0.18(0.08-0.33)	1.41(0.67-2.55)	0.06(0.03-0.1)	-3.18 (-3.39~-2.96)
Pakistan	121.92(55.33-201.54)	0.21(0.1-0.35)	202.79(118.32-301.1)	0.18(0.1-0.26)	-0.96 (-1.17~-0.74)
Palau	0.01(0-0.01)	0.07(0.04-0.1)	0.01(0.01-0.02)	0.05(0.03-0.07)	-1.04 (-1.16~-0.92)
Palestine	2.5(0.83-4.77)	0.33(0.1-0.64)	1.61(0.98-2.19)	0.08(0.04-0.11)	-5.48 (-5.95~-5.00)
Panama	1.53(1.16-2.24)	0.09(0.07-0.13)	2.78(1.69-4.55)	0.07(0.04-0.11)	-1.39 (-1.72~-1.06)
Papua New Guinea	5.83(2.53-11.5)	0.31(0.13-0.64)	9.99(4.84-17.87)	0.2(0.1-0.37)	-1.38 (-1.45~-1.32)
Paraguay	6.46(3.91-9.04)	0.26(0.16-0.37)	9.79(6.21-14.66)	0.16(0.1-0.24)	-2.37 (-2.68~-2.06)
Peru	12.12(7.43-17.94)	0.1(0.06-0.15)	12.6(8.35-20.51)	0.04(0.03-0.06)	-3.39 (-3.72~-3.05)
Philippines	25.36(18.1-41.48)	0.1(0.06-0.13)	148.1(88.99-207.34)	0.17(0.1-0.23)	2.94 (2.15~3.72)
Poland	1101.76(833.68-1342.48)	2.62(1.98-3.18)	1754.05(1196.57-2300.35)	2.78(1.89-3.65)	1.52 (0.85~2.20)
Portugal	56.82(46.81-71.1)	0.43(0.35-0.54)	66.49(43.51-86.23)	0.34(0.23-0.44)	0.10 (-0.90~1.12)
Puerto Rico	37.95(27.21-56.88)	1.06(0.76-1.59)	20.54(13.81-31.27)	0.35(0.23-0.53)	-4.95 (-5.61~-4.29)
Qatar	0.33(0.2-0.52)	0.35(0.17-0.73)	1.03(0.54-1.76)	0.15(0.08-0.25)	-3.13 (-3.62~-2.64)
Republic of Korea	29.22(17.62-47.53)	0.09(0.06-0.15)	23.08(14.85-31.17)	0.03(0.02-0.04)	-4.38 (-4.75~-4.00)
Republic of Moldova	14.91(9.22-45.95)	0.32(0.2-0.98)	229.51(152.19-283.06)	4.21(2.82-5.21)	12.78 (11.19~14.40)
Romania	944.12(657.8-1188.05)	3.4(2.4-4.27)	642.5(438.4-931.22)	2.05(1.38-3.02)	-1.52 (-1.85~-1.19)
Russian Federation	17463.96(13227.27-28284.35)	10.03(7.69-15.81)	31854.96(23739.04-39015.62)	15.65(11.7-19.17)	1.44 (-0.32~3.24)
Rwanda	4.51(2.06-10.49)	0.13(0.06-0.32)	2.86(1-6.07)	0.04(0.01-0.09)	-5.37 (-5.91~-4.82)
Saint Kitts and Nevis	0.47(0.35-0.67)	1.39(1.03-1.97)	1.17(0.78-1.55)	1.6(1.05-2.09)	1.07 (0.41~1.75)
Saint Lucia	2(1.47-2.88)	2.26(1.66-3.26)	5.77(4.09-7.33)	2.63(1.85-3.33)	1.37 (0.82~1.92)
Saint Vincent and the Grenadines	0.39(0.32-0.57)	0.55(0.44-0.79)	1.74(1.25-2.16)	1.27(0.91-1.57)	3.41 (3.08~3.74)
Samoa	0.24(0.15-0.38)	0.27(0.17-0.44)	0.25(0.15-0.4)	0.17(0.1-0.27)	-1.77 (-1.89~-1.64)
San Marino	0.41(0.28-0.56)	1.27(0.86-1.69)	0.54(0.33-0.85)	0.89(0.52-1.4)	-1.35 (-1.49~-1.21)
Sao Tome and Principe	0.25(0.13-0.42)	0.36(0.18-0.6)	0.25(0.13-0.42)	0.2(0.11-0.33)	-2.89 (-3.30~-2.48)
Saudi Arabia	13.17(6.72-27.4)	0.28(0.13-0.61)	14.88(8.98-22.72)	0.1(0.05-0.16)	-2.87 (-3.14~-2.59)
Senegal	13.14(6.84-22.15)	0.37(0.19-0.62)	10.98(5.45-19.74)	0.13(0.07-0.23)	-4.01 (-4.25~-3.77)
Serbia	91.77(54.13-158.71)	0.76(0.45-1.32)	150.19(81.59-245.45)	1.14(0.6-1.82)	2.06 (1.63~2.49)
Seychelles	0.53(0.34-0.75)	0.96(0.62-1.36)	0.76(0.5-1.01)	0.62(0.42-0.83)	-2.05 (-2.26~-1.83)
Sierra Leone	11.22(6.09-19.04)	0.57(0.31-0.97)	8.81(4.37-15.84)	0.22(0.11-0.39)	-3.85 (-4.12~-3.58)
Singapore	17.79(13.88-25.25)	0.68(0.54-0.91)	8.06(4.66-13.29)	0.1(0.06-0.17)	-7.69 (-8.99~-6.37)
Slovakia	42.46(31.79-61.11)	0.74(0.55-1.06)	139.45(69.99-202.99)	1.65(0.81-2.38)	5.13 (4.08~6.19)
Slovenia	317.07(119.85-569.19)	13.03(4.92-23.42)	9.22(5.14-13.76)	0.18(0.11-0.26)	-14.47 (-16.73~-12.15)
Solomon Islands	0.66(0.26-1.24)	0.44(0.19-0.76)	0.91(0.37-1.81)	0.27(0.12-0.48)	-1.54 (-1.64~-1.43)
Somalia	2.5(1.02-6.26)	0.08(0.03-0.19)	4.88(1.45-12.84)	0.06(0.02-0.17)	-0.94 (-1.00~-0.88)
South Africa	9.35(7.28-12.29)	0.04(0.03-0.05)	12.17(8.78-16.86)	0.03(0.02-0.04)	-1.32 (-1.61~-1.03)
South Sudan	1.11(0.36-2.22)	0.04(0.01-0.08)	1.1(0.4-2.24)	0.02(0.01-0.05)	-2.12 (-2.30~-1.94)

Spain	459.02(297.44-541.97)	0.89(0.58-1.05)	248.47(181.38-344.67)	0.31(0.22-0.43)	-4.07 (-4.43~-3.71)
Sri Lanka	74.25(45.53-110.6)	0.59(0.36-0.88)	209.74(101.03-347.94)	0.8(0.38-1.33)	2.20 (1.59~2.81)
Sudan	54.59(18.53-112.87)	0.6(0.19-1.26)	40.31(21.28-67)	0.21(0.11-0.35)	-3.90 (-4.01~-3.79)
Suriname	0.57(0.4-0.8)	0.2(0.14-0.28)	1.46(0.88-2.01)	0.23(0.14-0.32)	0.94 (0.44~1.44)
Sweden	103.83(83.2-133.5)	0.84(0.67-1.1)	124.3(91.57-162.03)	0.72(0.54-0.91)	-0.19 (-0.52~0.14)
Switzerland	170.74(122.12-208.93)	1.74(1.24-2.1)	78.39(52.54-106.03)	0.49(0.33-0.66)	-5.40 (-5.83~-4.97)
Syrian Arab Republic	17.07(9.64-27.13)	0.33(0.18-0.53)	18.8(8.29-31.52)	0.16(0.07-0.27)	-3.00 (-3.29~-2.71)
Taiwan (Province of China)	67.08(56.23-85.93)	0.43(0.35-0.53)	66.2(44.93-92.42)	0.19(0.13-0.26)	-3.91 (-4.32~-3.48)
Tajikistan	0.95(0.6-1.74)	0.03(0.02-0.05)	1.87(1.27-3.42)	0.03(0.02-0.05)	0.03 (-0.15~0.21)
Thailand	19.68(11.53-35.91)	0.05(0.03-0.1)	26.5(17.04-42.49)	0.03(0.02-0.04)	-2.78 (-3.00~-2.57)
Timor-Leste	0.44(0.21-0.89)	0.13(0.07-0.25)	1.01(0.53-1.92)	0.13(0.07-0.23)	-0.07 (-0.33~0.19)
Togo	6.21(3.42-10.18)	0.43(0.23-0.7)	7.64(4.28-13.31)	0.17(0.1-0.3)	-3.68 (-3.96~-3.40)
Tokelau	0(0-0.01)	0.3(0.15-0.5)	0(00)	0.13(0.07-0.19)	-3.12 (-3.27~-2.96)
Tonga	0.07(0.04-0.12)	0.13(0.07-0.21)	0.07(0.04-0.12)	0.09(0.05-0.15)	-1.24 (-1.35~-1.13)
Trinidad and Tobago	5.11(3.32-8.78)	0.58(0.38-1.01)	11.33(7.43-18)	0.6(0.39-0.94)	0.34 (0.05~0.63)
Tunisia	8.96(5.05-14.08)	0.19(0.11-0.3)	12.59(5.51-21.58)	0.1(0.05-0.17)	-2.26 (-2.34~-2.18)
Turkey	35.73(17.8-59.29)	0.1(0.05-0.16)	27.17(18.46-43.3)	0.03(0.02-0.05)	-4.45 (-4.69~-4.20)
Turkmenistan	25.43(12.95-54.5)	1.18(0.6-2.51)	71.22(36.38-158.28)	1.51(0.77-3.32)	0.39 (-0.02~0.79)
Tuvalu	0.03(0.01-0.05)	0.42(0.21-0.76)	0.02(0.01-0.03)	0.19(0.11-0.3)	-2.74 (-2.99~-2.48)
Uganda	5.52(2.34-10.72)	0.07(0.03-0.15)	7.59(3.21-14.66)	0.04(0.02-0.08)	-2.64 (-2.96~-2.32)
Ukraine	7836.82(6204.44-9731.22)	12.21(9.84-14.88)	10075.25(7730.14-14002.07)	16.24(12.44-22.27)	0.94 (0.48~1.41)
United Arab Emirates	2.28(0.77-4.87)	0.4(0.13-0.97)	9.03(2.99-18.08)	0.14(0.04-0.3)	-3.85 (-3.96~-3.73)
United Kingdom	455.22(394.11-594.25)	0.6(0.52-0.74)	488.79(396.83-627.51)	0.49(0.4-0.61)	-1.57 (-1.96~-1.18)
United Republic of Tanzania	7.34(3.15-14.13)	0.06(0.02-0.12)	13.02(4.25-24.37)	0.04(0.01-0.08)	-1.21 (-1.39~-1.02)
United States of America	6451.53(4570.24-7802.33)	2.13(1.52-2.57)	5825.56(4733.09-7349.54)	1.13(0.93-1.4)	-2.92 (-3.30~-2.54)
United States Virgin Islands	2.64(1.93-3.89)	2.8(2.04-4.16)	5.45(3.58-7.2)	3.04(2-4.01)	1.50 (0.66~2.35)
Uruguay	88.38(65.68-124.46)	2.3(1.72-3.25)	50.2(36.42-69.81)	1(0.72-1.37)	-4.07 (-4.61~-3.53)
Uzbekistan	4.67(1.82-8.33)	0.04(0.01-0.07)	10.72(7.94-16.85)	0.05(0.04-0.07)	1.35 (1.14~1.56)
Vanuatu	0.26(0.12-0.43)	0.38(0.18-0.64)	0.45(0.23-0.73)	0.25(0.13-0.42)	-1.66 (-1.86~-1.46)
Venezuela (Bolivarian Republic of)	99.03(63.43-146.23)	0.99(0.63-1.53)	83.74(56.36-118.74)	0.27(0.18-0.39)	-6.37 (-7.15~-5.58)
Viet Nam	57.44(29.5-106.4)	0.14(0.07-0.26)	155.6(87.92-288.33)	0.16(0.09-0.28)	0.78 (0.56~1.00)
Yemen	25.62(8.06-56.22)	0.57(0.17-1.27)	29.29(15.83-47.1)	0.23(0.11-0.37)	-3.75 (-4.05~-3.44)
Zambia	1.25(0.67-2.79)	0.04(0.02-0.08)	3.36(1.76-6.71)	0.04(0.02-0.08)	-0.06 (-0.22~0.10)

ACM, alcoholic cardiomyopathy; ASDR, age-standardized death rate; EAPC: estimated annual percentage change.

Table S2. The DALYs and age-standardized DALY rate of ACM in 1990 and 2019, and its temporal trends in 204 countries and territories from 1990 to 2019

Countries and territories	1990		2019		1990-2019
	DALYs cases No.(95%UI)	age-standardized DALY rate per 100,000 No. (95%UI)	DALYs cases No. (95%UI)	age-standardized DALY rate per 100,000No. (95%UI)	EAPC No. (95%UI)
Afghanistan	1589.25(370.05-3206.46)	20.75(4.79-41.59)	1628.12(516.99-2814.13)	9.81(2.87-17.68)	-3.10 (-3.63~-2.58)
Albania	744.77(550.46-1044.21)	32.18(23.67-44.19)	735.47(427.1-1357.99)	19.61(11.11-36.02)	-1.54 (-1.79~-1.29)
Algeria	790.31(479.92-1199.95)	5.64(3.32-8.66)	1052.05(542.34-1671.93)	2.84(1.44-4.54)	-2.51 (-2.65~-2.37)
American Samoa	4.98(3.09-6.9)	16.16(10.26-22.37)	2.33(1.63-3.57)	4.42(3.13-6.65)	-5.45 (-5.91~-4.98)
Andorra	31.57(20.34-47.61)	53.2(34.52-80.71)	40.89(28.51-56.58)	30.38(21.53-42.1)	-2.24 (-2.37~-2.11)
Angola	228.8(101.26-402.93)	4.17(1.97-7.43)	463.29(281.74-737.96)	2.79(1.68-4.47)	-1.40 (-1.48~-1.32)
Antigua and Barbuda	3.87(2.89-5.7)	7.89(5.85-11.67)	15.84(9.11-21.46)	14.36(8.32-19.29)	3.21 (2.57~3.84)
Argentina	10965.74(7946.01-14661.9)	33.78(24.63-45.03)	2607.26(1963.18-3394.36)	5.21(3.92-6.83)	-8.20 (-9.01~-7.39)
Armenia	3449.35(2672.18-4555.54)	121.06(96.21-150.97)	1199.04(797.71-2042.53)	31.23(20.91-53.06)	-5.36 (-6.43~-4.28)
Australia	10636.6(8844.51-12574.73)	56.61(46.89-66.88)	11730.97(8737.92-15025.41)	33.8(25.14-42.85)	-2.04 (-2.32~-1.77)
Austria	987.68(746.23-2156.4)	9.11(6.98-18.9)	3077(2078.48-4340.71)	19.68(12.95-28.01)	2.03 (0.78~3.30)
Azerbaijan	1040.59(479.32-2473.52)	18.15(8.4-43.59)	1746.07(761.88-4762.83)	15.29(6.92-40.94)	-1.31 (-1.68~-0.95)
Bahamas	104.3(78.53-145.62)	55.55(41.85-78.14)	298.95(213.7-391.2)	67.53(48.47-88.62)	1.67 (1.14~2.20)
Bahrain	21.28(14.76-32.7)	9.58(6.41-16.39)	54.47(32.86-91.01)	5.33(3.41-7.85)	-2.29 (-2.71~-1.87)
Bangladesh	3408.9(1258.49-5906.44)	5.76(2.28-9.87)	5390.41(2650.7-9029.84)	3.81(1.89-6.28)	-1.29 (-1.41~-1.17)
Barbados	110.2(87.45-143.65)	44.87(35.61-58.22)	238.41(171.95-305.37)	53.76(38.58-68.77)	1.14 (0.85~1.43)
Belarus	25731.62(17599.98-36062.36)	213.74(147.11-299.81)	40884.5(27838.84-55426.72)	323.82(220.53-437.92)	1.47 (1.08~1.87)
Belgium	2971.52(2339.05-3534.81)	21.37(17.11-24.97)	3041.97(2206.81-3958.49)	16.46(11.77-21.7)	-1.36 (-1.86~-0.86)
Belize	9.75(5.55-15.75)	9.4(5.38-15.08)	54.88(36.2-69.31)	16.39(10.87-20.7)	3.11 (2.66~3.57)
Benin	275.92(155.15-443.28)	12.55(6.92-20.41)	300.33(177-503.49)	4.85(2.86-8.22)	-3.92 (-4.30~-3.54)
Bermuda	6.46(4.03-11.74)	9.92(6.18-18.07)	12.34(7.45-16.5)	11.07(6.71-14.82)	1.22 (0.53~1.91)
Bhutan	15.58(5.85-30.35)	5.1(1.92-9.94)	21.61(12.94-34.9)	3.5(2.13-5.65)	-1.52 (-1.61~-1.42)
Bolivia	187.35(87.46-321.78)	5.25(2.4-9.41)	227.76(138.24-337.73)	2.41(1.48-3.52)	-2.63 (-2.95~-2.31)
Bosnia and Herzegovina	2028.31(1061.3-3173.75)	47.75(25.07-74.29)	2139.2(980.68-3714.91)	40.29(17.7-70.15)	-0.58 (-0.82~-0.34)
Botswana	13.39(8.81-21.41)	1.67(1.11-2.58)	23.16(13.81-36.49)	1.17(0.71-1.8)	-2.06 (-2.53~-1.60)
Brazil	64221.89(49657.56-80376.46)	55.92(43.19-70.37)	44396.81(34106.29-54733.25)	17.84(13.73-21.97)	-5.11 (-5.58~-4.63)
Brunei Darussalam	135.07(90.11-199.11)	74.46(51.05-108.08)	110.52(81.82-156.89)	25.23(18.84-35.18)	-4.10 (-4.35~-3.85)
Bulgaria	718.27(417.96-1062.84)	6.6(3.94-9.44)	354.91(242.75-555.62)	3.42(2.37-5.03)	-5.47 (-7.60~-3.28)

Burkina Faso	889.58(501.23-1531.52)	17.58(9.89-30.42)	844.9(492.21-1459)	7.49(4.35-12.99)	-3.53 (-3.91~-3.14)
Burundi	166.31(70.47-371.6)	5.64(2.4-12.72)	136.19(67.73-255.61)	2.13(1.08-3.97)	-4.20 (-4.61~-3.78)
Cabo Verde	5.76(4.05-8.24)	2.45(1.7-3.57)	13.46(9.28-19.29)	2.77(1.91-3.93)	-0.12 (-0.30~0.07)
Cambodia	256.98(108.31-567.14)	4.32(1.89-9.37)	473.73(271.03-967.62)	3.34(1.96-6.73)	-0.98 (-1.11~-0.84)
Cameroon	803.86(434.33-1386.33)	14.48(7.81-24.94)	1106.09(651.03-1807.66)	6.9(4.03-11.38)	-3.02 (-3.26~-2.78)
Canada	10153.9(8620.58-11928.91)	32.28(27.58-38.14)	12406.38(9662.97-15307.1)	21.4(17-26.37)	-1.63 (-1.83~-1.44)
Central African Republic	97.23(38.61-181.8)	6.25(2.7-11.78)	138.25(57.4-267.44)	4.42(2.02-8.69)	-1.30 (-1.39~-1.21)
Chad	402.67(209.89-732.44)	13.11(6.67-23.99)	442.27(235.06-790.12)	6.24(3.28-11.14)	-3.10 (-3.41~-2.79)
Chile	3355.35(2508.15-4088.17)	30.22(22.75-36.82)	1866.17(1388.98-2393.12)	8.2(6.11-10.48)	-4.88 (-5.42~-4.33)
China	35534.74(20068.92-70319.01)	3.41(1.89-6.89)	87822.91(57879.08-117542.45)	4.56(3.01-6.06)	2.18 (1.50~2.87)
Colombia	2437.12(1750.9-2775.8)	10.96(7.82-12.53)	568.79(384.95-871.32)	1.09(0.74-1.66)	-8.31 (-9.86~-6.73)
Comoros	4.02(1.45-7.95)	1.54(0.6-3.02)	5.71(2.16-10.74)	0.99(0.39-1.87)	-1.89 (-2.29~-1.50)
Congo	65.99(26.76-119.14)	4.79(2.11-8.48)	87.32(49.47-145.5)	2.37(1.36-3.85)	-2.67 (-2.91~-2.43)
Cook Islands	0.45(0.29-0.64)	3.02(1.95-4.21)	0.3(0.2-0.42)	1.42(0.94-1.98)	-2.97 (-3.28~-2.65)
Costa Rica	249.97(187.06-316.25)	11.81(8.72-15.07)	371.85(248.84-524.6)	7.06(4.72-9.99)	-2.78 (-3.61~-1.94)
Croatia	1899(1556.86-2862)	31.64(26.26-46.53)	4601.78(3171.85-6287.35)	60.19(40.83-82.72)	0.10 (-1.74~1.97)
Cuba	3151.9(2595.76-4398.24)	30.25(25.07-42.23)	13795.28(10289.52-17370.57)	78.85(58.48-98.99)	4.86 (4.24~5.48)
Cyprus	123.73(77.27-179.04)	14.8(9.05-21.54)	118.01(85.4-165.32)	6.41(4.67-9.09)	-3.34 (-3.63~-3.06)
Czechia	466.08(357.27-803.76)	3.79(2.9-6.56)	2997.25(1789.4-4055.05)	19.32(11.33-26.28)	6.02 (5.18~6.87)
C 么 te d'Ivoire	977.96(531.13-1620.12)	17.95(9.79-29.74)	920.17(557.05-1440.2)	6.41(3.92-10)	-4.50 (-4.94~-4.07)
Democratic People's Republic of Korea	1372.19(669.02-2813.28)	7.07(3.47-14.35)	2378.78(1471.56-4117.42)	7.39(4.6-13.07)	0.30 (0.20~0.40)
Democratic Republic of the Congo	720.25(386.62-1235.93)	3.48(1.91-6.04)	1230.69(578.23-2513.77)	2.46(1.1-4.97)	-1.22 (-1.37~-1.07)
Denmark	1605.29(1223.02-2023.55)	24.03(18.14-29.98)	1236.47(932.05-1506.11)	13.58(10.13-16.36)	-2.48 (-3.09~-1.87)
Djibouti	3.66(1.49-7.14)	1.58(0.67-3.07)	10.63(3.81-20.92)	1.21(0.47-2.27)	-1.06 (-1.15~-0.97)
Dominica	10.94(7.62-16.02)	17.64(12.23-25.83)	21.42(13.5-30.43)	24.85(15.58-34.98)	1.88 (1.41~2.35)
Dominican Republic	356.8(250.46-525.41)	7.57(5.19-11.41)	1494.44(855.35-2195.2)	14.69(8.35-21.45)	3.17 (2.88~3.46)
Ecuador	131.98(91.3-198.48)	2.06(1.41-3.02)	173.84(123.88-241.95)	1.07(0.77-1.49)	-2.30 (-2.75~-1.84)
Egypt	2818.04(1547.07-4444.14)	7.89(4.08-12.83)	4135.25(2219.61-6845.15)	5.23(2.71-8.89)	-1.00 (-1.17~-0.83)
El Salvador	55.44(35.82-74.79)	1.6(1.03-2.18)	45.23(29.92-64.08)	0.74(0.49-1.06)	-3.39 (-3.84~-2.95)
Equatorial Guinea	14.28(5.57-27.68)	5.7(2.34-10.88)	14.39(7.2-25.99)	1.85(0.94-3.39)	-4.43 (-5.08~-3.77)
Eritrea	29.15(11.67-64.64)	1.92(0.85-4.27)	48.66(23.93-92.67)	1.17(0.61-2.15)	-1.87 (-2.03~-1.71)
Estonia	2749.85(1911.59-3764.44)	143.13(99.8-193.92)	2782.01(1928.12-	142.51(99.48-190.51)	0.10 (-0.84~1.06)

			3700.87)		
Eswatini	6.4(4.25-9.77)	1.48(0.99-2.2)	9.88(5.61-16.55)	1.15(0.69-1.89)	-0.79 (-1.10~-0.48)
Ethiopia	480.76(200.32-1178.32)	1.78(0.77-4.34)	503.37(227.17-873.9)	0.87(0.41-1.53)	-2.83 (-2.97~-2.69)
Fiji	9.56(6.7-13.64)	1.72(1.22-2.47)	10.3(7-14.59)	1.17(0.8-1.65)	-1.57 (-1.80~-1.34)
Finland	8046.17(5410.92-10294.97)	126.67(83.85-162.91)	6770.72(4970.68-7769.17)	79.49(55.76-91.26)	-1.34 (-1.70~-0.98)
France	23960.64(19490.55-27864.46)	33.06(26.96-38.07)	13925.96(9532.27-18743.73)	13.67(9.21-18.15)	-3.58 (-4.11~-3.05)
Gabon	29.91(13.03-59.19)	4.67(2.08-9.19)	32.66(19.07-52.09)	2.42(1.43-3.86)	-2.26 (-2.36~-2.15)
Gambia	58.66(31.2-100.99)	13.8(7.19-23.79)	69.13(40.39-111.63)	5.98(3.52-9.8)	-3.57 (-3.92~-3.21)
Georgia	289.87(158.47-422.32)	4.94(2.64-7.13)	384.69(235.99-775.3)	8.84(5.42-16.84)	2.82 (2.45~3.19)
Germany	133330.12(97892.45-165149.78)	117.25(85.28-144.19)	85210.39(68970.78-98805.87)	54.94(44.4-63.48)	-2.68 (-2.99~-2.38)
Ghana	1700.21(1034.03-2604.07)	21.71(13.29-33.28)	4334.29(2540.23-6551.57)	21.27(12.37-32.48)	0.10 (-0.15~0.35)
Greece	494.62(358.97-748.06)	3.74(2.72-5.59)	282.96(214.56-523.26)	1.9(1.45-3.51)	-2.37 (-2.60~-2.15)
Greenland	24.26(16.55-34.53)	48.74(34.05-68.7)	29.64(18.49-41.65)	39.1(24.7-54.01)	-0.08 (-0.62~0.47)
Grenada	28.98(20.95-43.68)	46.28(33.55-69.98)	72.06(44.44-90.96)	59.44(36.71-75.26)	1.77 (1.32~2.22)
Guam	15.18(10.79-20.23)	15.97(11.27-21.55)	7.09(4.82-11.57)	3.7(2.57-6.03)	-5.94 (-6.31~-5.58)
Guatemala	163.52(103.04-246.48)	3.66(2.24-5.58)	204.58(134.1-271.47)	1.56(1-2.1)	-3.40 (-3.91~-2.89)
Guinea	420.53(202.93-783.74)	11.51(5.46-21.51)	373.13(215.15-627.82)	5.64(3.23-9.61)	-2.64 (-2.87~-2.40)
Guinea-Bissau	100.73(38.14-209.49)	21.04(8.07-43.75)	71.7(41.64-122.47)	7.56(4.33-12.97)	-3.94 (-4.21~-3.66)
Guyana	455.76(353.47-564.9)	94.41(73.85-116.74)	511.91(324.22-712.7)	69.72(44.2-96.71)	0.29 (-0.75~1.36)
Haiti	3318.86(1328.15-5463.1)	82.09(34.94-137.38)	4394.17(2165.42-7491.05)	49.59(25.33-85.14)	-1.47 (-1.75~-1.18)
Honduras	408.09(209.32-608.36)	16.43(8.32-24.31)	766.96(363.51-1185.28)	11.44(5.47-17.35)	-1.29 (-1.45~-1.12)
Hungary	24895.38(21043.71-31504.46)	188.95(157.37-236.89)	35414.1(25084.39-45552.74)	227.42(159.1-292.09)	0.09 (-0.44~0.61)
Iceland	23.22(18.15-28.89)	8.64(6.77-10.72)	19.42(15.39-24.11)	3.95(3.15-4.88)	-2.98 (-3.17~-2.80)
India	31939.1(14588.46-52039.68)	5.85(2.7-9.73)	47929(33219.28-67159.05)	3.82(2.65-5.35)	-1.57 (-1.65~-1.48)
Indonesia	6249.2(3654.15-10026.63)	4.77(2.75-7.57)	10939.28(6079.98-20642.97)	4.17(2.35-7.69)	-0.09 (-0.23~0.06)
Iran (Islamic Republic of)	1850.44(1353.03-2864.94)	5.6(4.06-8.56)	2970.94(2082.48-3870.09)	3.57(2.44-4.66)	-1.31 (-1.58~-1.03)
Iraq	277.49(155.4-486.93)	3.08(1.66-5.69)	339.68(240.42-475.99)	1.17(0.83-1.66)	-4.16 (-4.53~-3.79)
Ireland	1324.55(1128.52-1732.2)	34.95(29.8-45.81)	1352.94(940.45-1836.17)	20.23(14.25-27.31)	-2.84 (-3.33~-2.34)
Israel	284.1(188.77-367.44)	6.15(4.14-8.05)	151.14(102.43-203.67)	1.51(1.04-2.04)	-6.32 (-7.91~-4.70)
Italy	19041.24(15456.47-31154.57)	24.22(19.85-37.79)	3723.89(2596.08-5740.53)	3.77(2.44-5.69)	-8.93 (-10.00~-7.84)
Jamaica	121.53(84.35-191.53)	7.18(4.9-11.49)	480.54(334.82-696.1)	16.06(11.2-23.23)	4.06 (3.39~4.73)
Japan	15578.37(12056.29-18467.54)	9.61(7.54-11.46)	9248.25(6689.32-	4.83(3.43-6.08)	-2.87 (-3.03~-2.72)

			11574.37)		
Jordan	43.67(24.67-64.03)	2.29(1.33-3.37)	93.29(68.62-124.52)	0.99(0.73-1.33)	-3.70 (-4.03~-3.37)
Kazakhstan	1205.92(472.29-4162.31)	8.45(3.33-29.12)	7834.63(2974.07-14588.52)	39.3(14.85-73.51)	7.85 (5.58~10.18)
Kenya	122.83(56.4-216.03)	1.12(0.52-1.99)	321.96(156.87-527.82)	1(0.5-1.65)	-0.36 (-0.51~-0.21)
Kiribati	4.4(2.11-6.65)	9.11(4.78-13.4)	6.49(2.94-10.34)	7.09(3.34-10.91)	-0.66 (-0.95~-0.37)
Kuwait	25.12(20.22-32.53)	2.3(1.82-3.13)	48.18(33.49-63.19)	1.23(0.87-1.61)	-2.39 (-2.93~-1.84)
Kyrgyzstan	5176.93(3589.31-6033.37)	155.8(107.85-181.96)	14432.35(6943.56-17983.15)	241.44(117.45-301.23)	2.35 (1.77~2.93)
Lao People's Democratic Republic	212.52(71.95-435.45)	8.29(2.86-16.94)	290(163.78-519.1)	4.95(2.85-8.8)	-1.83 (-1.91~-1.75)
Latvia	3801.34(2882.37-5677.81)	118.78(90.48-175.31)	16000.24(10794.77-20617.76)	629.33(428.39-813.26)	8.13 (6.65~9.63)
Lebanon	184.48(114.08-272.46)	7.27(4.51-10.66)	206.02(92.56-327.37)	3.96(1.78-6.29)	-2.17 (-2.31~-2.03)
Lesotho	17.36(9.84-29.48)	1.44(0.82-2.47)	22.47(13.51-37.89)	1.36(0.84-2.25)	0.32 (0.12~0.52)
Liberia	196.98(109.58-332.38)	16.39(9.06-27.8)	151.9(71.95-289.39)	5.52(2.68-10.51)	-4.44 (-4.89~-3.99)
Libya	107.36(58.48-171.12)	4.3(2.32-7.05)	194.95(99.7-298.61)	3(1.51-4.56)	-1.29 (-1.47~-1.10)
Lithuania	5094.89(4100.5-6461.19)	121.62(97.74-153.48)	6845.1(5035.6-8948.26)	178.4(131.77-232.56)	2.08 (0.49~3.69)
Luxembourg	177.46(137.01-212.84)	34.68(26.67-41.74)	129.67(83.85-176.63)	14.09(9.04-19.17)	-3.58 (-3.75~-3.41)
Madagascar	195.67(106.21-340.88)	2.76(1.48-4.84)	356.77(150.3-634.03)	2.04(0.88-3.62)	-1.14 (-1.20~-1.08)
Malawi	106.76(58.15-191.4)	2.16(1.17-3.92)	177.33(76.58-320.22)	1.78(0.8-3.19)	-0.89 (-1.06~-0.73)
Malaysia	226(166.96-321.28)	1.96(1.43-2.93)	307.93(216.1-426.48)	1(0.71-1.38)	-2.77 (-3.01~-2.53)
Maldives	4.41(1.82-7.79)	3.64(1.43-6.45)	5.63(2.84-8.08)	1.2(0.64-1.7)	-4.21 (-4.62~-3.80)
Mali	469.5(266.7-867.2)	9.62(5.45-17.86)	436.3(228.08-757.45)	3.98(2.06-6.88)	-3.49 (-3.92~-3.06)
Malta	48.42(37.93-58.56)	11.34(8.9-13.74)	42.06(27.67-54.49)	5.51(3.59-7.16)	-2.68 (-2.84~-2.52)
Marshall Islands	2.32(1.03-3.96)	11.33(5.39-19.53)	3.02(1.36-5.04)	6.92(3.34-11.26)	-1.77 (-1.82~-1.72)
Mauritania	144.98(78.23-252.79)	12.83(6.85-22.41)	85.96(34.19-161.88)	3.47(1.42-6.47)	-4.74 (-5.11~-4.36)
Mauritius	145.53(102.46-206.5)	14.23(10.11-19.88)	135.32(94.2-186.84)	8.94(6.19-12.45)	-2.45 (-3.52~-1.36)
Mexico	2578.95(2130.7-3341.58)	4.74(3.87-6.09)	4655.03(3513.49-6456.83)	3.66(2.77-5.06)	-0.56 (-0.91~-0.21)
Micronesia (Federated States of)	6.67(2.67-12.14)	11.59(4.99-21.38)	5.79(2.43-10.83)	6.64(2.88-11.97)	-2.08 (-2.32~-1.84)
Monaco	12.91(8.65-18.03)	24.52(16.5-34.08)	8.7(5.91-12.23)	12.44(8.54-17.63)	-2.50 (-2.69~-2.31)
Mongolia	356.99(185.48-704.53)	27.81(14.42-55.17)	1548.3(910.45-2922.04)	41.39(24.51-77.57)	1.42 (1.28~1.55)
Montenegro	1831.15(929.5-2842.48)	293.22(145.7-459.95)	2107.98(1114.97-3176.36)	229.01(122.03-342.65)	-0.96 (-1.26~-0.65)
Morocco	1575.25(729.27-2919.54)	10.31(4.72-19.71)	1610.81(896.51-2406.93)	4.73(2.63-7.01)	-2.91 (-3.02~-2.80)
Mozambique	162.32(67.84-335.15)	2.18(0.93-4.46)	327.81(170.1-648.85)	2.19(1.15-4.33)	0.32 (0.18~0.46)
Myanmar	2131.74(848.86-4194.79)	7.19(3.05-13.84)	2117.43(1311-3651.57)	3.92(2.46-6.67)	-2.23 (-2.30~-2.15)
Namibia	12.24(7.6-19.05)	1.37(0.85-2.14)	20.11(12.79-31.95)	1.08(0.72-1.69)	-1.05 (-1.23~-0.88)
Nauru	0.6(0.26-1.27)	10.17(4.81-20.03)	0.48(0.21-1.03)	6.79(3.26-13.74)	-1.40 (-1.78~-1.02)
Nepal	632.59(292.46-1093.96)	5.6(2.73-9.63)	959.65(555.32-1568.02)	3.98(2.29-6.5)	-1.24 (-1.38~-1.10)

Netherlands	9543.93(7657.87-11068.44)	50.82(41.13-58.83)	4282.8(2687.89-6212.21)	14.85(9.13-21.63)	-5.19 (-5.58~-4.79)
New Zealand	2528.12(1785.74-3507.82)	68.62(48.23-95.45)	2435.26(1684.07-2808.01)	37.58(25.91-43.32)	-2.45 (-2.82~-2.09)
Nicaragua	73.89(52.41-99.49)	3.71(2.59-5.01)	95.89(67.92-129.83)	1.9(1.36-2.53)	-2.67 (-3.09~-2.24)
Niger	453.74(238.18-866.47)	13.09(6.85-25.06)	486.07(238.95-908.32)	4.79(2.34-9.08)	-3.91 (-4.26~-3.57)
Nigeria	11194.88(5793.72-18437.35)	21.34(11.1-35.29)	6529.02(3784.12-10390.89)	5.52(3.25-8.66)	-5.64 (-6.14~-5.14)
Niue	0.14(0.09-0.21)	6.53(4.15-9.88)	0.08(0.05-0.12)	3.84(2.33-5.86)	-2.18 (-2.34~-2.02)
North Macedonia	1568.53(1166.56-2069.1)	88.59(64.29-118)	484.62(258.51-779.42)	16.54(9.05-26.44)	-7.87 (-8.94~-6.79)
Northern Mariana Islands	7.17(4.58-10.32)	26.35(17.3-37.26)	3.73(2.51-5.85)	5.95(4.15-9.54)	-5.81 (-6.30~-5.32)
Norway	1065.33(848.36-1199.9)	20.84(16.61-23.29)	573.8(456.29-756.31)	7.67(6.13-9.91)	-3.31 (-3.69~-2.93)
Oman	42.67(21.78-77.82)	4.68(2.24-8.62)	57.52(26.13-105.42)	1.79(0.95-3.01)	-2.94 (-3.18~-2.69)
Pakistan	3537(1547.04-5980.92)	5.53(2.52-9.19)	6610.04(3737.82-10029.15)	4.78(2.79-7.05)	-0.82 (-1.04~-0.59)
Palau	0.29(0.18-0.46)	2.24(1.44-3.41)	0.41(0.26-0.57)	1.79(1.18-2.54)	-0.74 (-0.84~-0.64)
Palestine	53.46(22.02-97.31)	5.97(2.14-11.11)	50.95(34.95-65.4)	1.73(1.1-2.3)	-4.59 (-4.96~-4.22)
Panama	61.61(47.03-91.2)	3.27(2.48-4.88)	111.94(69.14-184.4)	2.63(1.63-4.32)	-1.07 (-1.42~-0.73)
Papua New Guinea	200.35(85.61-400.11)	8.23(3.65-16.17)	351.69(165.26-615.41)	5.55(2.75-9.81)	-1.28 (-1.34~-1.22)
Paraguay	233.63(139.86-323.3)	8.81(5.33-12.27)	350.71(224.14-516.13)	5.59(3.58-8.27)	-2.28 (-2.58~-1.97)
Peru	365.34(228.64-541.95)	2.64(1.64-3.89)	393.55(265.4-613.65)	1.18(0.8-1.84)	-2.91 (-3.27~-2.55)
Philippines	1002.58(739.46-1710.6)	2.55(1.84-4.03)	5874.52(3579.62-8342.81)	5.84(3.54-8.19)	4.05 (2.97~5.14)
Poland	27396.3(23199.27-33423)	64.01(54.22-78.15)	50185.41(34166.66-65566.42)	85.69(58.19-112.32)	2.27 (1.66~2.90)
Portugal	1673.45(1427.45-2104.07)	13.35(11.22-16.82)	1903.49(1281.21-2391.52)	11.11(7.56-14.01)	0.07 (-0.85~1.00)
Puerto Rico	1124.92(819.34-1619.19)	31.38(22.85-44.96)	610(415.14-914.74)	11.39(7.82-16.67)	-4.55 (-5.15~-3.94)
Qatar	12.91(8.27-19.29)	6.95(4.06-12.1)	48.34(27.93-78.79)	2.91(1.71-4.48)	-3.36 (-3.63~-3.09)
Republic of Korea	1074.84(669.01-1748.13)	2.76(1.7-4.48)	707.44(499.72-920.58)	0.95(0.69-1.24)	-3.82 (-4.22~-3.42)
Republic of Moldova	568.54(365.47-1623.31)	12.19(7.85-34.66)	7691.33(5284.9-9527.92)	147.14(101.08-184.32)	12.32 (10.78~13.88)
Romania	32618.17(23041.46-40604.98)	119.46(84.02-147.82)	19632.96(13239.23-28929.49)	67.57(45.32-98.66)	-1.89 (-2.22~-1.56)
Russian Federation	625037.73(459849.8-1004631.46)	359.48(265.49-571.29)	1158387.46(875859.18-1420501.96)	602.19(452.33-738.45)	1.61 (-0.24~3.49)
Rwanda	174.22(80.98-403.39)	4.64(2.16-10.56)	120.31(49.8-218.65)	1.5(0.65-2.84)	-5.02 (-5.58~-4.46)
Saint Kitts and Nevis	14.18(10.6-20.26)	43.69(32.36-63.31)	35.86(23.84-48.95)	46.61(30.9-63.26)	0.73 (0.07~1.40)
Saint Lucia	62.52(46.89-88.66)	69.08(51.59-97.09)	179.3(129.55-226.92)	80.82(58.24-101.97)	1.39 (0.87~1.91)
Saint Vincent and the Grenadines	12.52(10.07-17.57)	17.19(13.9-24.32)	53.14(38.07-66.64)	38.65(27.75-48.15)	3.28 (2.97~3.58)
Samoa	7.33(4.56-11.53)	7.24(4.56-11.3)	7.92(4.83-12.54)	4.75(2.9-7.44)	-1.54 (-1.63~-1.46)
San Marino	10.47(7.55-13.66)	33.72(24.42-43.82)	12.74(7.81-19.76)	23.56(14.38-36.82)	-1.39 (-1.54~-1.24)
Sao Tome and Principe	8.22(4.35-13.5)	11.75(6.23-19.26)	9.22(5.04-15.41)	6.81(3.77-11.22)	-2.71 (-3.11~-2.30)
Saudi Arabia	335.28(192.69-619.69)	5.29(2.77-10.74)	500.01(310.94-722.8)	2.2(1.31-3.27)	-2.50 (-2.75~-2.26)

Senegal	438.52(241.48-728.17)	11.85(6.46-19.85)	403.45(209.34-692.91)	4.43(2.35-7.68)	-3.77 (-4.02~-3.52)
Serbia	3207.95(1959.35-5080.85)	27.08(16.53-42.28)	4754.04(2505.18-7477.69)	37.98(19.31-60.43)	1.74 (1.35~2.13)
Seychelles	17.2(11.3-24.23)	31.26(20.46-44.35)	25.73(17.15-34.33)	20.33(13.69-26.72)	-2.05 (-2.28~-1.82)
Sierra Leone	370.32(208.14-614.69)	18.13(10.19-30.48)	328.11(172.16-576.28)	7.41(3.85-13.05)	-3.60 (-3.86~-3.34)
Singapore	671.39(511.47-1029.9)	22.32(17.39-32.1)	282.48(178.68-428.83)	3.63(2.35-5.47)	-7.40 (-8.56~-6.23)
Slovakia	1541.25(1177.38-2169.98)	27.07(20.4-38.17)	4492.56(2260.31-6484.38)	55.83(27.65-81.25)	4.75 (3.77~5.74)
Slovenia	5986.23(2834.28-9809.53)	245.28(117.51-401.86)	170.69(123.14-236.17)	3.97(2.84-5.65)	-14.07 (-16.34~-11.74)
Solomon Islands	23.99(8.39-49.17)	12.84(5.04-24.27)	33.13(12-71.73)	7.9(3.26-15.74)	-1.53 (-1.65~-1.42)
Somalia	99.83(43.81-243.74)	2.69(1.22-6.42)	201.06(72.96-486.17)	2.1(0.79-5.31)	-0.93 (-0.99~-0.87)
South Africa	388.24(306.54-505.95)	1.29(1.02-1.67)	468.52(335.48-650.35)	0.88(0.64-1.22)	-1.56 (-1.81~-1.32)
South Sudan	43.2(16.68-84.11)	1.44(0.56-2.81)	46.29(19.65-87.69)	0.87(0.4-1.64)	-1.87 (-2.04~-1.70)
Spain	10568.38(8279.37-12587.57)	21.23(16.91-25.4)	6991.19(5207.54-9782.56)	9.33(7.02-13.28)	-3.30 (-3.53~-3.07)
Sri Lanka	2755.43(1732.33-4045.57)	20.16(12.64-29.58)	6997.88(3408.06-11524.75)	26.77(13.16-44.02)	1.99 (1.42~2.56)
Sudan	1654.54(575.75-3296.97)	15.53(5.41-31.5)	1306.94(696.37-2143.55)	5.57(3.02-9.21)	-3.80 (-3.91~-3.69)
Suriname	19.37(13.53-27.21)	6.43(4.55-9.05)	47.78(29.55-65.61)	7.4(4.54-10.11)	0.89 (0.38~1.39)
Sweden	3355.01(2749.47-4334.68)	29.55(24.08-38.99)	3706.65(2903.1-4556.77)	23.68(18.65-29.01)	-0.49 (-0.76~-0.21)
Switzerland	4426.6(3187.22-5313.39)	47.67(34.23-57.14)	2013.46(1421.58-2651.55)	13.56(9.58-17.92)	-5.32 (-5.73~-4.91)
Syrian Arab Republic	511.34(314.75-786.81)	8.11(4.67-12.41)	591.48(284.68-981.06)	4.26(2.07-6.98)	-2.70 (-2.99~-2.41)
Taiwan (Province of China)	2316.64(1976.85-2943.31)	12.5(10.66-15.69)	2444.66(1690.5-3388.96)	7.44(5.13-10.32)	-2.51 (-2.79~-2.23)
Tajikistan	37.07(23.15-66.81)	1.01(0.61-1.93)	75.92(52.75-133.75)	0.99(0.69-1.76)	-0.43 (-0.67~-0.19)
Thailand	688.36(433.54-1175.32)	1.56(0.97-2.72)	910.65(623.81-1386.54)	0.98(0.68-1.46)	-2.12 (-2.31~-1.92)
Timor-Leste	17.23(8.27-36.73)	3.82(1.9-7.56)	36.09(18.26-68.87)	3.91(2.01-7.43)	0.17 (-0.13~0.47)
Togo	213.04(122.85-344.24)	13.74(7.7-22.21)	280.02(166.36-477.98)	5.79(3.43-9.77)	-3.48 (-3.75~-3.20)
Tokelau	0.11(0.06-0.18)	8.1(4.25-13.57)	0.05(0.03-0.07)	3.62(2.08-5.61)	-2.92 (-3.06~-2.78)
Tonga	2.2(1.29-3.53)	3.57(2.11-5.69)	2.21(1.26-3.58)	2.66(1.52-4.29)	-1.00 (-1.08~-0.92)
Trinidad and Tobago	154.68(103.1-263.42)	17.13(11.3-29.64)	338.24(223.81-532.87)	18.07(11.98-28.38)	0.47 (0.19~0.76)
Tunisia	267.41(157.35-414.78)	4.72(2.75-7.26)	377.17(183.45-627.91)	2.93(1.46-4.84)	-1.79 (-1.86~-1.72)
Turkey	1161.57(661.08-1808.8)	2.7(1.46-4.31)	874.28(616.48-1261.19)	0.95(0.68-1.37)	-4.21 (-4.47~-3.95)
Turkmenistan	940.86(491.4-1985.47)	40.17(20.3-85.32)	2724.69(1401.88-6050.99)	54.44(27.86-120.51)	0.62 (0.22~1.02)
Tuvalu	0.88(0.41-1.53)	11.52(5.49-19.81)	0.6(0.35-0.95)	5.47(3.21-8.52)	-2.53 (-2.78~-2.28)
Uganda	208.95(94.33-390.03)	2.54(1.15-4.69)	319.26(144.54-574.29)	1.54(0.73-2.84)	-2.43 (-2.76~-2.10)
Ukraine	273474.65(216734.3-334296.41)	432.39(345.47-522.6)	371846.87(283126.56-517522.89)	636.34(483.52-875.78)	1.34 (0.85~1.83)

United Arab Emirates	91.41(32.65-181.06)	10.59(3.55-24.43)	383.42(138.02-722.27)	4.1(1.51-8.2)	-3.42 (-3.57~-3.26)
United Kingdom	14666.49(12801.89-18060.18)	21.06(18.27-26.2)	15523.49(12542.29-18777.06)	17.16(13.55-20.6)	-1.54 (-1.92~-1.17)
United Republic of Tanzania	279.78(135-508.55)	2.06(0.99-3.76)	531.18(204.22-937.21)	1.62(0.65-2.86)	-1.00 (-1.17~-0.83)
United States of America	193909.09(144716.14-229200.56)	67.36(50.42-79.43)	174248.54(146468.47-212903.51)	36.05(30.76-44.05)	-2.76 (-3.16~-2.35)
United States Virgin Islands	87.36(64.81-128.37)	86.56(63.92-127.44)	150.56(99.48-198.64)	90.49(59.46-122)	1.23 (0.48~1.99)
Uruguay	2324.92(1736.54-3234.32)	62.42(46.62-86.94)	1317.5(962.7-1763.48)	28.07(20.54-37.49)	-3.95 (-4.48~-3.42)
Uzbekistan	153.07(68.32-274.29)	1.09(0.46-2.02)	381.08(279.18-622.74)	1.39(1.05-2.11)	1.13 (0.97~1.29)
Vanuatu	8.62(3.77-14.52)	10.27(4.77-17.1)	15.1(7.24-25.73)	7.22(3.7-11.86)	-1.55 (-1.77~-1.34)
Venezuela (Bolivarian Republic of)	3001.16(1941.72-3993.83)	26.89(17.22-37.56)	2720(1783.05-3876.4)	8.68(5.76-12.44)	-5.55 (-6.24~-4.85)
Viet Nam	1913.84(1023.88-3611.14)	4.2(2.22-7.96)	5586.35(3078.12-10553.33)	5.1(2.89-9.49)	1.27 (1.01~1.53)
Yemen	754.69(248.68-1604.21)	13.46(4.3-29.15)	912.38(518.77-1439.06)	5.62(3.09-9.01)	-3.53 (-3.82~-3.23)
Zambia	49.63(28.03-103.1)	1.29(0.75-2.76)	138.75(76.51-254.55)	1.41(0.79-2.63)	0.05 (-0.10~0.20)
Zimbabwe	70.29(46.06-93.91)	1.05(0.69-1.44)	144.24(84.9-217.03)	1.24(0.75-1.82)	0.81 (0.69~0.94)

ACM, alcoholic cardiomyopathy; DALY: disability-adjusted life year; EAPC: estimated annual percentage change.

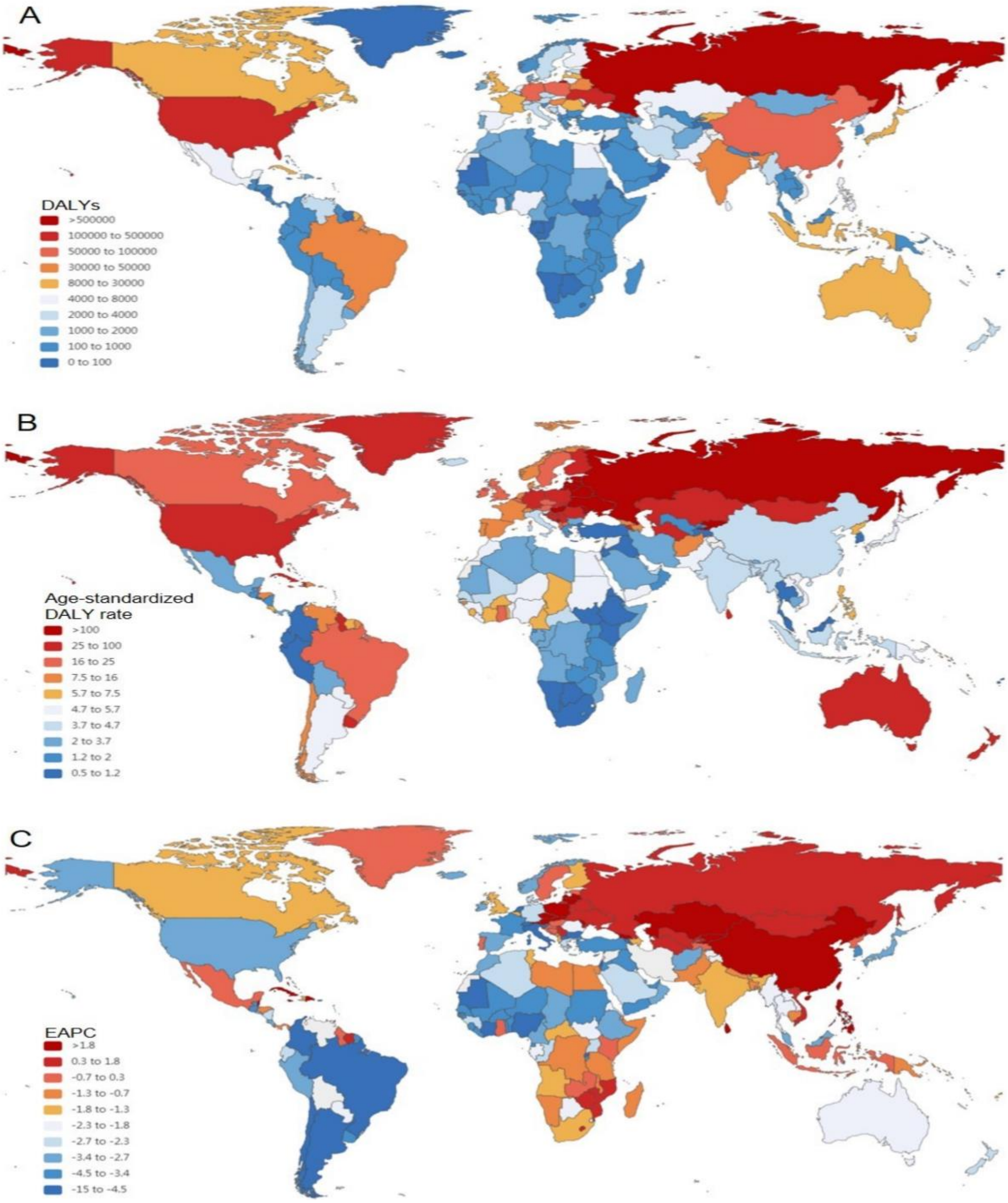

Figure S1. The global DALYs burden of ACM in 204 countries and territories. **Panel A**. The absolute number of ACM DALYs in 2019; **Panel B**. The ACM age standardized DALY rate (per 100,000 persons) in 2019; **Panel C**. The EAPC of alcoholic cardiomyopathy age standardized DALY rate between 1990 and 2019. ACM, alcoholic cardiomyopathy; DALY: disability-adjusted life year; EAPC, estimated annual percentage change.

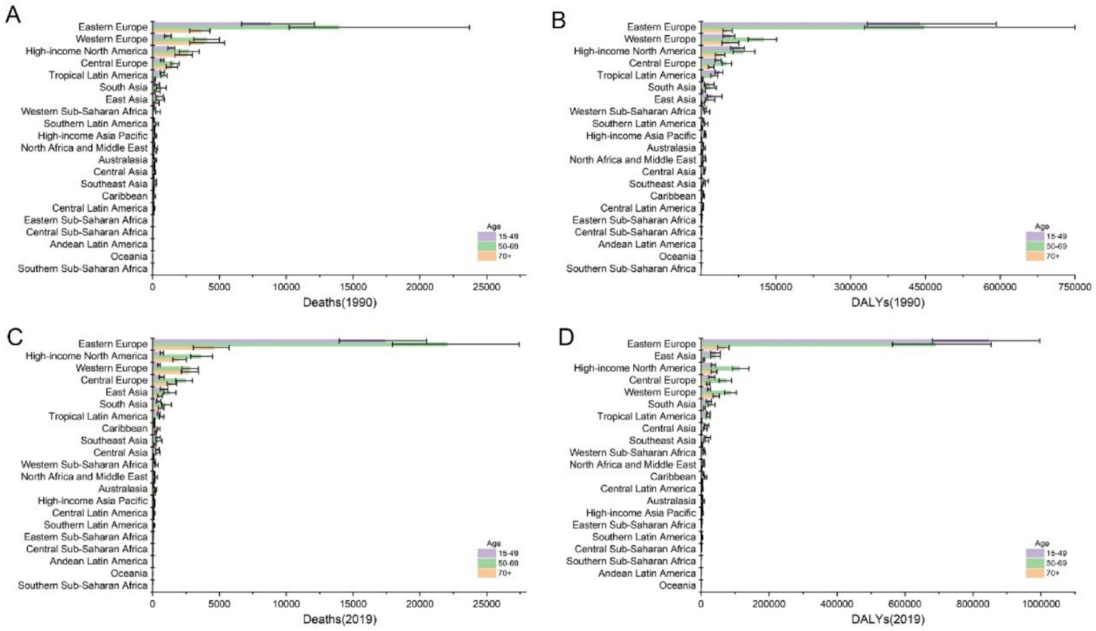

Figure S2. The proportion of the three age groups (15-49 years, 50-69 years and 70+ years) for ACM deaths and DALYs in 21 GBD regions in 1990 (**Panel A** and **Panel B**) and 2019 (**Panel C** and **Panel D**). ACM, alcoholic cardiomyopathy; DALY: disability-adjusted life year; GBD: global burden of disease.
